# Copper-binding ligands in deep-sea pore waters of the Pacific Ocean and potential impacts of polymetallic nodule mining on the copper cycle

**DOI:** 10.1038/s41598-021-97813-3

**Published:** 2021-09-16

**Authors:** Sophie A. L. Paul, Rebecca Zitoun, Ann Noowong, Mythili Manirajah, Andrea Koschinsky

**Affiliations:** 1 Department of Physics and Earth Sciences, Jacobs University Bremen, Campus Ring 1, 28759 Bremen, Germany; 2 GEOMAR Helmholtz Centre for Ocean Research Kiel, Wischhofstr. 1-3, 24148 Kiel, Germany; 3 Department of Ocean Systems (OCS), Utrecht University, Royal Netherlands Institute for Sea Research (NIOZ), 1797 SH ‘t Horntje, The Netherlands

**Keywords:** Biogeochemistry, Ocean sciences

## Abstract

The release of potentially toxic metals, such as copper (Cu), into the water column is of concern during polymetallic nodule mining. The bioavailability and thus toxicity of Cu is strongly influenced by its speciation which is dominated by organic ligand (L) complexation in seawater, with L-complexes being considered less bioavailable than free Cu^2+^. The presence of CuL-complexes in deep-sea sediments has, however, not been systematically studied in the context of deep-sea mining. We thus analyzed the Cu-binding L concentration ([L]) in deep-sea pore waters of two polymetallic nodule provinces in the Pacific Ocean, the Peru Basin and the Clarion-Clipperton-Zone, using competitive ligand equilibration–adsorptive stripping voltammetry. The pore-water dissolved Cu concentration ([dCu]) ranged from 3 to 96 nM, generally exceeding bottom water concentrations (4–44 nM). Based on fitting results from ProMCC and Excel, Cu was predominantly complexed by L (3–313 nM) in bottom waters and undisturbed pore waters. We conclude that processes like deep-sea mining are unlikely to cause a release of toxic Cu^2+^ concentrations ([Cu^2+^]) to the seawater as > 99% Cu was organically complexed in pore waters and the [Cu^2+^] was < 6 pM for 8 of 9 samples. Moreover, the excess of L found especially in shallow pore waters implied that even with a Cu release through mining activities, Cu^2+^ likely remains beneath toxic thresholds.

## Introduction

The bioavailability and thus toxicity of dissolved Cu (dCu) is strongly influenced by its speciation (i.e. the distribution of Cu between chemical species) and complexation. Generally, free Cu^2+^ or weakly inorganically complexed Cu (CuX_IN_) such as CuOH or CuCl is considered more bioavailable (bioavailable Cu: Cu’ = Cu^2+^ + CuX_IN_) than Cu organically complexed by a ligand (L) (CuL)^1,2^. An organic ligand is an organic compound that binds to a central metal ion, such as Cu. The stable organic complexation keeps Cu in solution and thus inhibits biological uptake or adsorption to particles and thereby removal from the water column^3,4^, nevertheless, some organically complexed Cu can also be bioavailable^5,6^. In seawater, Cu complexation is largely controlled by L with commonly more than 99% of dCu bound to L^7^. Complexation depends on the availability of the natural organic L and is characterized by the thermodynamic stability of the metal–ligand complex (conditional stability constants, Log*K’*_*CuL, Cu2*+_)^3^. The Log*K’*_*CuL, Cu2*+_ are often divided into a stronger (L_1_) and a weaker ligand class (L_2_)^3^. Organic L are largely of biogenic origin and can be biologically produced by various marine organisms or result from the input or breakdown of organic substances, that is during microbial degradation^8–10^. Examples of organic Cu-binding L are marine humic substances derived from decaying phytoplankton and reduced sulfur substances derived from diagenetic reactions of sulfur with organic matter but many ligands are not yet well characterized^3,11–13^.

The release of bioavailable metals that might be toxic to benthic fauna, such as Cu, Cd, and Pb, is of concern in the context of deep-sea mining^14^. Specifically Cu has gained a lot of attention in the metal-ecotoxicology community due to its deleterious effects on living organisms at very low concentrations and has thus often been used as an example metal to study negative effects that a seafloor disturbance could have on prevalent biota^15–17^. Heavy metals could be increasingly released from pore water to bottom seawater in a disturbance event at the seafloor, such as polymetallic nodule mining, because the pore-water dissolved Cu concentration [dCu], especially in the surface pore water, is usually higher than in bottom seawater^18,19^. A removal of the upper cm of sediment due to mining activity would therefore lead to a sudden dCu release into the near-bottom seawater instead of the usual “slow” diffusion of various metals across the sediment-water-interface^18^. A recent study in the Peru Basin, a polymetallic nodule province, showed that 5 weeks after the removal of surface sediment, the signature [dCu] peak in the upper ca. 2 cm of pore water was absent, indicating that dCu had been released to the bottom water^18^. This release of dCu during experiments simulating mining disturbances raises the question about the fate of dCu and other bioactive metals after disturbance events and its potentially toxic effects on prevalent deep-sea fauna. Additionally, dCu could be adsorbed onto or desorbed from particles in the sediment plume that is expected to accompany mining of polymetallic nodules, thereby impacting the oceanic Cu cycle as well^20,21^. A study in San Francisco Bay found that the contribution of dCu desorbed from resuspended sedimentary particles is a much larger source to the water column than diffusion from pore waters^1,22^. Thus, understanding Cu complexation and speciation pre- and post-disturbance activities would help to assess the potential threat of Cu toxicity from released pore waters and suspended particles in the plume-affected water column.

Assessing Cu toxicity for benthic fauna is, however, not straightforward and studies rarely differentiate between Cu species and complexes while studying ecotoxicological effects. Additionally, toxicity depends strongly on the organism’s tolerance level. While a concentration of Cu^2+^ ([Cu^2+^]) as low as 1 pM can negatively affect cyanobacteria reproduction, eukaryotic plankton does not seem to be affected by 10 pM [Cu^2+^]^23^. Ecotoxicological experiments at atmospheric conditions with three shallow- and one deep-water hydrothermal vent shrimp species showed that up to 4 µM Cu (without specification of the studied Cu species) can be tolerated by adult shrimp species (sublethal concentrations) at surface pressure, but in one shallow-water species (*Palaemon elegans*) significant Cu increases were found in the tissue (gills and hepatopancreas)^24^. Sublethal and toxic effects might, however, be different in larvae or brooding females and at deep-sea environmental conditions (e.g., higher hydrostatic pressure, lower temperature, altered pH, complex metal mixtures)^24,25^. For instance, experiments with shallow-water shrimps and nematodes showed that colder temperatures reduce Cu toxicity, while higher hydrostatic pressure significantly increased Cu toxicity^15,17^. These Cu toxicity assessments with nematodes resulted in lethal concentrations where 50% of the organisms died (LC_50_ values) at ca. 8–28 µM dCu, depending on temperature, hydrostatic pressure, and exposure time^15^. However, none of these studies assessed Cu speciation and/or organic Cu complexation fully, which makes the toxicity assessments difficult to compare. Consequently, differences in Cu tolerance levels of various organisms, insufficient quantification of Cu speciation, and the ability of some organisms to produce organic ligands to mitigate Cu toxicity makes any generalizations about the effects of Cu in the marine environment challenging^8,26,27^. Thus, more research, especially on ecotoxicological effects in complex, multi-stressor environments, such as the deep-sea, is necessary.

While numerous studies exist for [dCu], Cu-binding L concentrations ([L]), and conditional stability constants (Log*K’*_*CuL, Cu2*+_) in the water column of the open ocean^3,7,28,29^, estuaries^30,31^, shallow^32^ and deep-sea hydrothermal vent sites^9,10,33^, studies focusing on pore-water dCu and Cu speciation parameters are rare across these environments. More than two decades ago, a study of Chesapeake Bay showed that organic Cu-binding L exist in estuarine pore waters and that these pore waters are a significant source of L to the water column^34^. Yet, Cu-binding L have not been analyzed in pore waters of the abyssal plains, even though the deep seafloor, because of its vastness, might be a large source of organic L to the water column.

Consequently, the current study presents dCu and Cu-binding L data of pore water and bottom seawater of three sites in the Pacific Ocean, namely the German and Belgian license areas for mining in the Clarion Clipperton Zone (CCZ) and the Peru Basin. Both the CCZ and the Peru Basin are polymetallic nodule provinces where environmental impacts of deep-sea mining have been extensively studied during the past decades^18,35,36^. Samples from 11 cores including the overlying multicorer (MUC)/push core (PUC) bottom water and one Niskin bottle sampled with remotely operated vehicle (ROV) were taken in 2015, 2018, and 2019. Samples included natural, undisturbed pore waters (8 cores), pore waters from 26-year-old plow tracks in the Peru Basin (2 cores), and 5-week-old plume-covered sediments in the German license area of the CCZ (1 core) to study impacts of polymetallic nodule mining on the overall Cu biogeochemistry.

## Study sites

**Figure 1:**
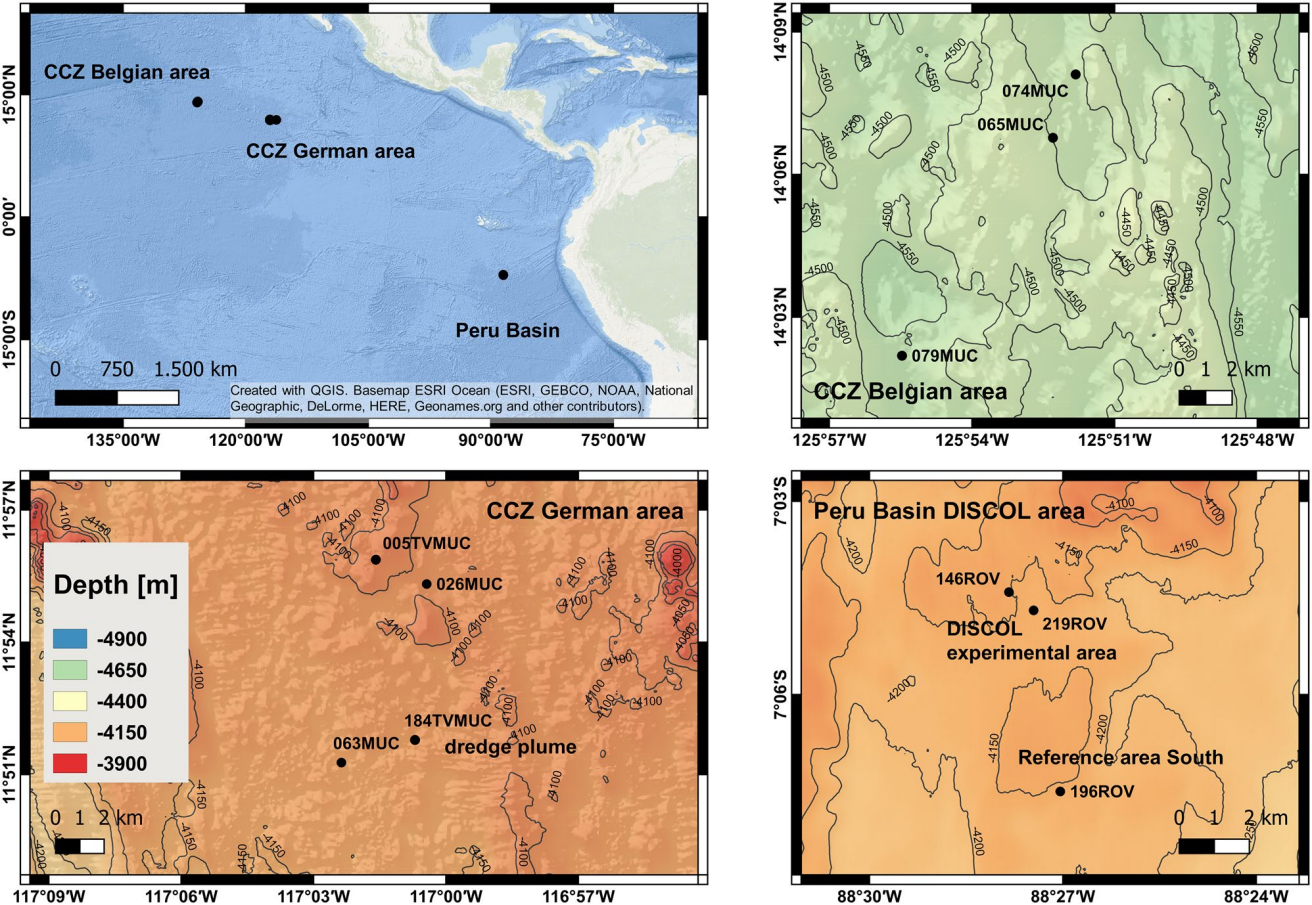
Sampling locations in the Belgian (top right) and German (bottom left) license areas for polymetallic nodule mining in the CCZ and in the DISCOL area in the Peru Basin (bottom right). 149MUC is not displayed in the CCZ German area map, because it is located further east. For details see Table 1. Close-up maps: data from EM 122 multibeam echosounder during SO242^45^ and SO268^46,47^.

The Peru Basin is located in the south-east equatorial Pacific Ocean, at the southern border of the equatorial high productivity zone and the coastal upwelling region of Ecuador. It receives comparatively higher organic matter inputs than the CCZ [compare^37,38^]. Water depths in this study area are between 4100 and 4250 m (Fig. 1). Total organic carbon (TOC) contents in the surface sediments are between 0.5 and 1 wt%^18,36^. Oxygen penetration depths are ca. 12–25 cm^18,36^ and suboxic conditions commence just below the lowest samples from the sediment cores presented here. After oxygen is consumed, denitrification and Mn-oxide reduction govern organic matter degradation^36^. Sediment core samples were collected in 2015 during expedition SO242 from one unimpacted reference site and from two 26-year-old plow tracks of the *DISturbance and reCOLonization in a manganese nodule area of the deep South Pacific Ocean* (DISCOL) experiment^39,40^. In 1989, the DISCOL project was carried out by plowing an 11 km^2^ large circular area on the deep seafloor, referred to as the DISCOL experimental area (DEA), using a custom-made “plow-harrow”^39,41^. The plow track samples had the labile organic-rich, reactive top-layer removed, mixed, or turned around with the associated plume blanketing the surface sediments^18,35,40,42,43^. The plow tracks in the DEA are not homogenous^44^ and show various disturbance features previously classified as microhabitats^18^. For the study presented here we sampled sediment cores in a “valley” – a small furrow within the plow tracks – and a “white patch” – a spot where the sediment has likely been turned upside down and the lighter colored, deeper sediment has been exposed at the surface. Both are typical disturbance features.

**Table 1:** Sample overview of sediment cores and one Niskin bottle sampled for Cu-binding L during SO242, SO262, and SO268. *bw* = bottom water.

Sample ID	Sampling depth ligand samples	Lat	Lon	Area	Water depth [m]
SO242/2 146ROV-Niskin	bw	7°04.4094′ S	88°27.8366′ W	Peru Basin: Disturbed site DEA West “valley”	4139
SO242/2 146ROV-PUC28	bw				
	2, 3, 4 cm				
	11, 12, 13 cm				
SO242/2 196ROV-PUC28	bw	7°07.5041′ S	88°27.0406′ W	Peru Basin: Undisturbed Reference South	4156
	3, 4, 5 cm				
	16, 17, 18 cm				
SO242/2 219ROV-PUC28	bw	7°04.6930′ S	88°27.4540′ W	Peru Basin: Disturbed site DEA South “white patch”	4155
	2, 3, 4 cm				
	10, 11, 12 cm				
SO262 026MUC	bw	11°55.320′ N	117°00.435′ W	CCZ German area undisturbed	4102
	2, 5, 8 cm				
	12, 15, 18 cm				
	21, 25, 27 cm				
SO262 063MUC	bw	11°51.271’ N	117°02.368’ W	CCZ German area undisturbed	4131
	1, 5.5, 8.5 cm				
	12, 15, 19 cm				
	23, 27, 30 cm				
SO262 149MUC	bw	11°53.833′ N	116°14.268′ W	CCZ German area undisturbed	4105
	1.5, 4.5, 8.5 cm				
	12.5, 15, 17.5 cm				
	21, 25, 30 cm				
SO268/1 005TVMUC	bw	11°55.872′ N	117°01.588′ W	CCZ German area undisturbed	4081
	2, 6, 9 cm				
	17, 20, 23 cm				
SO268/2 184TVMUC	bw	11°51.785′ N	117°00.701′ W	CCZ German area: Dredge plume deposition	4116
	2, 5, 8 cm				
	17, 20, 23 cm				
SO268/1 065MUC	bw	14°06.772′ N	125°52.297′ W	CCZ Belgian area undisturbed	4495
	2, 5, 8 cm				
	15, 18, 22 cm				
SO268/1 074MUC	bw	14°08.105′ N	125°51.819′ W	CCZ Belgian area undisturbed	4509
	3, 5, 9 cm				
	16, 19, 22 cm				
SO268/1 079MUC	bw	14° 02.187′ N	125° 55.471′ W	CCZ Belgian area undisturbed	4535
	3, 6, 9 cm				
	17, 20, 23 cm				

The three sampling depths that are given are the depths where rhizons were inserted and then pooled as one sample.

The CCZ is located in the central equatorial Pacific Ocean between the Clarion fracture to the North and the Clipperton fracture to the South. Water depths in the study area are between 4000 and 5000 m (Fig. 1). The area is characterized by low inputs of organic matter, even though this is variable across the CCZ, and surface sediment TOC contents are 0.2–0.6 wt%^37^. The conditions in pore waters in the CCZ study sites are oxic down to several meters depth^37^ and all surface (< 30 cm) samples presented here are thus from oxic pore waters. Organic matter degradation is predominantly governed by aerobic respiration, followed by denitrification and Mn-oxide reduction in deeper sediments^37^. Sediment core samples from undisturbed sites of the German (4 cores) and Belgian (3 cores) license areas for polymetallic nodule mining were taken in 2018 and 2019 during expeditions SO262 and SO268, respectively (Fig. 1). During SO268, one sediment core from a dredge disturbance in the German area (ca. 10 tracks) was also taken, ca. 5 weeks post-impact. This sediment core was, however, not collected from within a dredge track, but from an area impacted by the resettled sediment plume, south of the disturbed site and ca. 2 m away from the nearest dredge track.

## Results and discussion

### Total dCu measured in ligand samples

**Figure 2:**
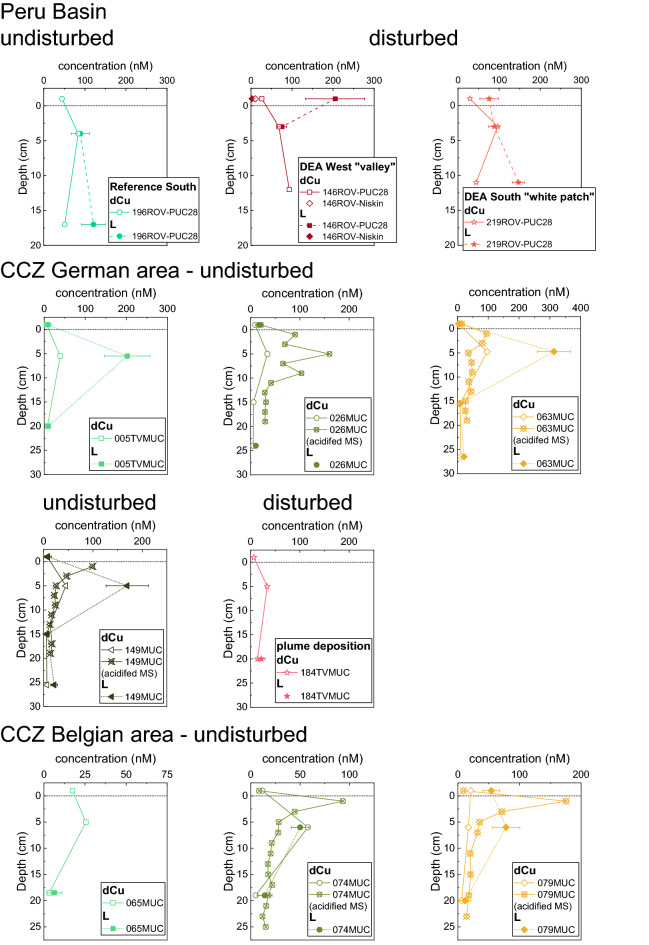
[dCu] and [L] plotted vs. depth for each core from the Peru Basin and CCZ—German and Belgian license areas (pooled samples, for details see Table 1). The uppermost value (− 1 cm) shows the bottom water. For some cores, [dCu] data from a second core sampled at higher resolution, acidified with HCl and measured by ICP-MS is shown. Because of pooling of the low-resolution dCu data for the ligand samples, the high- and low-resolution dCu profiles sometimes deviate with high-resolution data usually showing higher [dCu] in the upper few cm. For the pooled samples, the average depth is shown. Note different concentration axes scales. Bottom water for 065MUC was sampled from an adjacent liner and not from the same liner from which pore water was sampled.

At all sites, bottom water and two to three depths for pore water were sampled for dCu and L analyses. Some sites were also sampled at higher resolution (~ 2 cm) for dCu (Fig. 2). Unless specifically stated, we refer to the low resolution dCu and associated L data in the results and discussion.

[dCu] generally agreed with previously published concentrations for the Peru Basin^18,19^. Small differences in concentration and profile shape between published data and this data set can be explained by differences in sample treatment, including freezing, as is done for ligand analyses, compared to acidification and cold storage (also compare the [dCu] measurements in Fig. 2 for the pooled ligand samples and the higher resolution acidified samples) or by temporal variations of sampling seasons and years. For the CCZ area, no published data of pore-water [dCu] exist so far to the best of our knowledge.

**Figure 3:**
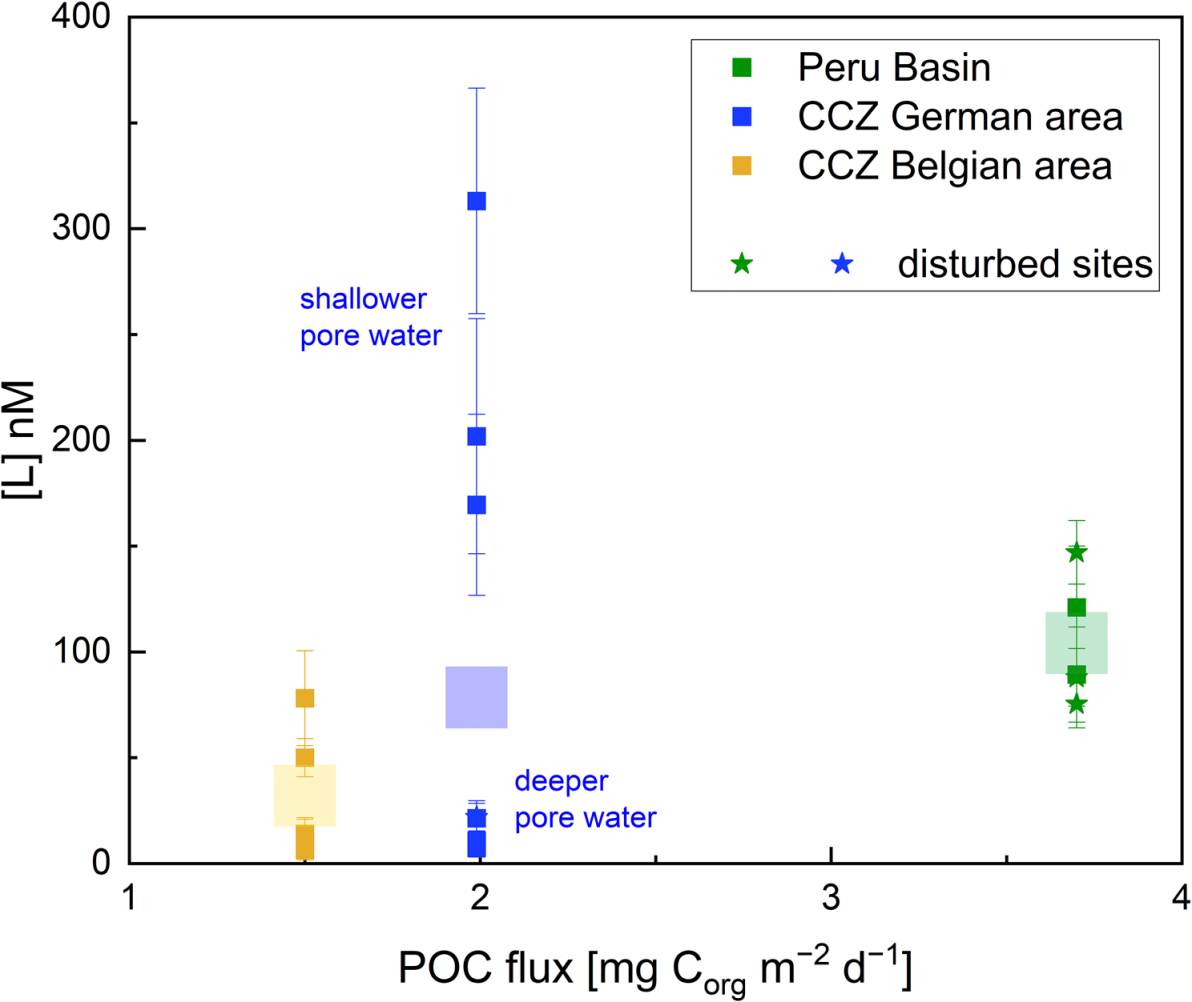
Relationship between [L] and POC flux for the three study sites. The relationship between organic carbon deposition on the seafloor and L can help to assess the potential of organic matter as a source of L. POC data from^37^ for the CCZ and^36^ for the Peru Basin. Only pore-water [L] is shown here. Note POC data were chosen and calculated for the general area, not the specific sampling locations of this study.

Total [dCu] of undisturbed bottom waters ranged from 3.8 to 44.4 nM (Fig. 2, Table 2), with highest concentrations in the Peru Basin. Concentrations of bottom waters at disturbed sites also fell within this range, suggesting that no impact of the disturbance on the total [dCu] is discernable in the time frame of our sampling (5 weeks and 26 years). At all sites except 79MUC in the CCZ Belgian area, [dCu] in surface pore waters shallower (<) 10 cm were higher than bottom water values, corroborating findings from other studies that pore waters are enriched in dCu compared to seawater owing to dCu release to pore waters during organic matter degradation^18,19,48,49^. [dCu] in undisturbed surface pore waters < 10 cm was variable between sites and ranged from 17–96 nM, with an average of 50 ± 26 nM. In deeper pore waters > 10 cm, [dCu] was lower than in the upper 10 cm at all undisturbed sites, ranging from 3 to 51 nM, with an average of 11 ± 13 nM. With depth, [dCu] presumably decreased as no dCu is released below the subsurface layer into the pore water due to less (labile) organic matter and microbial degradation. For both, the undisturbed shallower and deeper pore waters, average concentrations increased from the Belgian area, over the German area, to the Peru Basin (Fig. 2; Table 2). The higher [dCu] in the Peru Basin might be explained by the higher particulate organic carbon (POC) flux to the seafloor in the Peru Basin compared to the CCZ^36,37^ (Fig. 3). We hypothesize that the more organic matter reaches the seafloor, the more [dCu] is also released during organic matter degradation at the sediment-water-interface. However, the smaller sampling interval (2–4 cm) used in the Peru Basin might have affected [dCu], because it only targeted the subsurface peak while the pore water in the CCZ was pooled from the upper 10 cm of the core, where concentrations commonly decreased below ca. 4 cm. This decrease is illustrated in the higher resolution [dCu] profiles in Fig. 2. The subsurface peak is usually more elevated, if the sampling resolution is higher, capturing the upper 0.5 or 2 cm, as can be seen in the depth profiles of [dCu] from the higher resolution sampling (Fig. 2). The pooling conducted during sampling for this study to get sufficient pore water for ligand analyses likely obscures this maximum.

**Table 2:** Overview of [dCu], [L], Log*K’*_*CuL, Cu2*+_, [Cu^2+^], and [Cu’] for all samples.

Area	Sample ID	Sampling depths	[dCu] nM	[L] nM	Error [L] nM	Log*K’*_*CuL, Cu2*+_	Error Log*K’*_*CuL, Cu2*+_	[Cu^2+^] pM	[Cu’] pM
Peru Basin	SO242/2 146ROV-Niskin (disturbed)	bw	10.9	2.7*	1.7*	–	–	–	–
	SO242/2 146ROV-PUC28 (disturbed)	bw	26.6	205.2	72.0	11.97	0.3	0.48	12.5
		2, 3, 4 cm	67.9	75.4	11.3	12.47	0.3	3.00	78.1
		11, 12, 13 cm	93.3	–	–	–	–	–	–
	SO242/2 196ROV-PUC28	bw	44.4	–	–	–	–	–	–
	(undisturbed)	3, 4, 5 cm	83.9	89.3*	22.4*	–	–	–	–
		16, 17, 18 cm	50.9	121.0	29.0	12.04	0.2	0.67	17.4
	SO242/2 219ROV-PUC28 (disturbed)	bw	28.9	75.8*	22.8*	–	–	–	–
		2, 3, 4 cm	96.2	88.0	13.6	12.19	0.2	0.66	17.1
		10, 11, 12 cm	44.2	147.0	15.0	13.05	0.2	0.17	4.4
CCZ—German	SO262 026MUC	bw	9.5	17.3*	7.0*	–	–	–	–
	(undisturbed)	2, 5, 8 cm	34.2	–	–	–	–	–	–
		12, 15, 18 cm	6.1	–	–	–	–	–	–
		21, 25, 27 cm	5.3	10.8*	4.4*	–	–	–	–
	SO262 063MUC	bw	3.8	7.2*	5.3*	–	–	–	–
	(undisturbed)	1, 5.5, 8.5 cm	96.4	313.1	53.2	12.05	0.1	4.00	99.9
		12, 15, 19 cm	9.0	7.4*	4.4*	–	–	–	–
		23, 27, 30 cm	11.4	21.4*	6.9*	–	–	–	–
	SO262 149MUC	bw	7.3	8.7*	7.1*	–	–	–	–
	(undisturbed)	1.5, 4.5, 8.5 cm	45.1	169.5	42.8	11.80	0.1	5.68	142.0
		12.5, 15, 17.5 cm	5.6	7.4*	4.4*	–	–	–	–
		21, 25, 30 cm	6.8	21.4*	6.9*	–	–	–	–
	SO268/1 005TVMUC	bw	6.0	8.6*	8.1*	–	–	–	–
	(undisturbed)	2, 6, 9 cm	38.9	201.9	55.5	11.77	0.2	4.01	104.4
		17, 20, 23 cm	7.9	10.1*	5.3*	–	–	–	–
	SO268/2 184TVMUC	bw	6.8	–	–	–	–	–	–
	(disturbed)	2, 5, 8 cm	33.8	–	–	–	–	–	–
		17, 20, 23 cm	14.2	21.9*	7.8*	–	–	–	–
CCZ—Belgian	SO268/1 065MUC	bw	17.4	–	–	–	–	–	–
	(undisturbed)	2, 5, 8 cm	25.5	–	–	–	–	–	–
		15, 18, 22 cm	3.2^a^	6.4*	4.7*	–	–	–	–
	SO268/1 074MUC	bw	11.8	–	–	–	–	–	–
	(undisturbed)	3, 5, 9 cm	57.8	50.0	9.0	12.75	0.2	322.53	8385.9
		16, 19, 22 cm	4.8	14.1*	7.5*	–	–	–	–
	SO268/1 079MUC	bw	20.8	54.2	13.4	12.40	0.2	2.46	64.0
	(undisturbed)	3, 6, 9 cm	17.1	78.1	22.5	12.14	0.2	2.03	52.8
		17, 20, 23 cm	6.1	11.8*	9.1*	–	–	–	–

[L_T_] (L_T_ = total L, sum of L_1_ and L_2_) are highlighted with a star (*) and indicate samples that could not be fitted with the ProMCC software due to an insufficient amount of titration points for the reliable use of the program. These samples were thus fitted with the linest function in Excel (linear fitting using the least squares method). Excel does not produce Log*K’*_*CuL, Cu2*+_, Cu^2+^ and Cu’ estimates. Bioavailable Cu: Cu’ = Cu^2+^ + CuX_IN_. bw = bottom water.– = no data. All samples except the bottom water were pooled from three depths as listed in the sampling depths.

^a^This sample is just below the LOD for the ICP-MS run. For details on LOD see “Methods”.

At the disturbed sites < 10 cm, pore-water [dCu] was lowest in the core from the plume deposition site in the German license area (34 nM) and highest in the Peru Basin 26-year-old plow tracks (82 ± 14 nM) (Fig. 2, Table 2). Deeper pore waters > 10 cm from disturbed sites showed [dCu] of 14 nM at the German CCZ plume covered site and 69 ± 25 nM at the DEA in the Peru Basin. Concentrations were variable and no clear difference between undisturbed and disturbed sites was detectable, which could also be related to the time frame of sampling (5 weeks and 26 years) and that the pore waters, especially in the Peru Basin, already regained a new equilibrium after 26 years^18^.

### Copper-binding ligand fitting

The concentration of complexing [L], corresponding conditional stability constants (Log*K’*_*CuL, Cu2*+_), as well as [Cu^2+^] and bioavailable [Cu’] were determined using the ProMCC^50^ fitting software. Titration data were difficult to fit in the ProMCC software^50^ because of the low resolution of data points below and in the linear section of the titration curve owing to pore-water volume restrictions of titrated samples in this pilot study. Consequently, no fit could be produced for 26 samples out of 37 samples. For the 11 remaining samples, the fitted data could be explained by a one-ligand model. Data that could not be fitted in ProMCC was fitted with the least squares method in Excel and resulted in 17 more results for the total L concentration ([L_T_] = L_1_ + L_2_). For 9 samples no reliable fit could be produced with any method. Even though the fitting was complicated, the overall trends are reliable, also because of consistent data fitting throughout.

### Copper complexation in bottom waters

Cu-binding organic [L] in deep-sea bottom waters were between 3 and 205 nM. Lowest average [L] in bottom water were found in the CCZ German license area followed by the Belgian license area (Table 2). No [L] bottom water data was available for the Peru Basin undisturbed site. [L] in bottom water at disturbed sites was exceptionally high with 205 nM in the PUC bottom water of the DEA West "valley" track. However, the Niskin sample from the DEA West track only had a [L] of 3 nM (Fig. 2, Table 2). Seawater [L] down to 3000 m depth are lower than measured in our study, usually < 5 nM^3,28,51^, except for our Niskin [L] of 3 nM. Very little data exists to our knowledge for deeper waters and recent data from the Pacific showed [L] up to 9 nM in water depths between 4000–5000 m^29^. The authors hypothesized that sediments might be a source of L, leading to the higher near-bottom [L]^29^.

**Figure 4:**
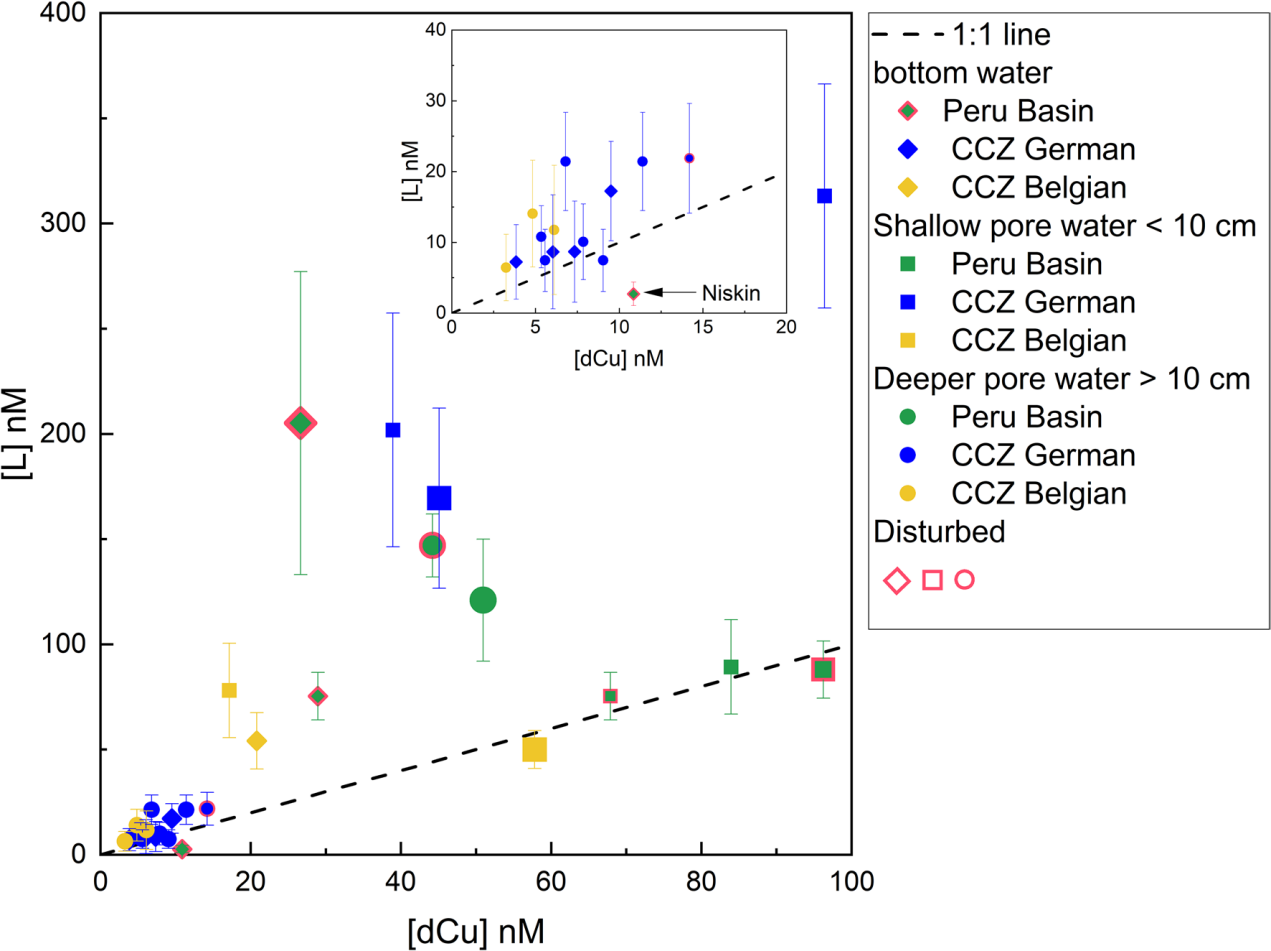
[dCu] vs. [L] of all samples for which [L] could be calculated (28 of 37 samples, 11 with ProMCC and 17 with Excel). ). Samples with a good ProMCC fit, that are most reliable, are plotted as bigger symbols. In samples that plot above the 1:1 line, L are present in excess of dCu and all Cu is presumably complexed by L. All samples plot within error range of the 1:1 line or above, except the ROV-Niskin sample from the Peru Basin.

Based on the few bottom water data points from our study (only 8 of 12 could be fitted, of which only 2 could be fitted with ProMCC), it is not clear which impact mining has on the bottom water Cu complexation by L. Within the error range, [L] was in excess of [dCu] in bottom waters indicating almost exclusively complexation as CuL in bottom waters except for the Niskin bottom water sample, where [dCu] was in excess of [L] (Fig. 4). CuL was > 99% for the one undisturbed core from the Belgian area that could be fitted and [Cu^2+^] was correspondingly low with 2.46 pM. Bottom water of undisturbed and disturbed sites showed no clear difference in [L]. However, [L] in both cases by far exceeded [dCu], meaning that even higher [Cu^2+^] released by future mining activities can potentially be buffered by the ambient [L] and that toxicity thresholds for prevalent biota will be potentially not exceeded. Nevertheless, bottom water sampled within hours of a disturbance and analyzed for [L] and [Cu^2+^] would be necessary to assess short-term impacts on benthic fauna.

When comparing bottom water from the ROV-PUC with bottom water from the ROV-Niskin, sampled ca. 1 m above the seafloor in the Peru Basin (146ROV), it is striking that [dCu] was elevated by a factor of 2.5 in the ROV-PUC bottom water, but [L] was elevated by a factor of 75. MUC and PUC bottom water can be impacted by sampling or interact with the sediment on the way up through the water column during instrument recovery. Deploying and retrieving the gear might also lead to a disturbance of the sediment surface and a release of dCu and L from the comparatively enriched pore waters. Such an impact could explain why the bottom water concentrations from the PUC sample had higher [L] and [dCu] values more similar to those of shallow pore waters. Thus, the potential for sampling artifacts needs to be considered and a complimentary assessment of [L] in the bottom water column using a specific water sampling method such as a bottom water sampler equipped with Niskins that samples within the lower 5 m of the water column is crucial in future studies. Nevertheless, the different composition of the Niskin sample could also reveal that the seawater [dCu] and [L] are already considerably altered a few tens of cm above the sediment-water-interface. This alteration may be due to quick dilution of the benthic flux of dCu and L by ambient bottom water. Unfortunately, the Niskin sample could not be fitted with ProMCC but the undisturbed MUC bottom water Log*K’*_*CuL, Cu2*+_ value for the one sample from the CCZ Belgian area that could be fitted (12.40) was slightly lower than published CLE-AdCSV seawater values for the Pacific Ocean water column, e.g., LogK 12.7–14.1 for the subarctic North Pacific Ocean^28^, LogK_1_ 15.0–16.5 and LogK_2_ 11.6–13.6 for the North East Pacific Ocean^3^, and LogK 12.5–14.0 for the South East Pacific Ocean^51^. For deep waters between 4000–5000 m, recent data from the Pacific displayed LogK of ca. 13.5–14.5^29^. The L sources in the near-bottom seawater are clearly different as indicated by our lower Log*K’*_*CuL, Cu2*+_ (12.40) compared to water column LogK from other studies, likely owing to diffusion from the sediment pore waters, as has been previously suggested^29^. These seem to have a slightly different composition of ligands than seawater, potentially due to different sources.

### Copper complexation in undisturbed deep-sea pore waters and potential ligand sources

[L] depth profiles generally followed [dCu] and [L] were usually enriched in the surface pore waters < 10 cm. [L] in undisturbed surface pore waters < 10 cm was between 50 and 313 nM, (Fig. 2, Table 2). On average, surface pore-water [L] was lowest in the Belgian license area, followed by the Peru Basin and the German license area (Fig. 2, Table 2). With depth, [L] generally decreased and in undisturbed pore waters > 10 cm depth, [L] ranged from 6–121 nM. [L] was considerably lower in the German and Belgian CCZ areas, with 6–21 nM, while the Peru Basin showed higher [L] with 121 nM. The [L] difference between sites could be related to the POC flux. The Belgian area has the lowest surface water primary productivity and lowest POC flux compared to the German area and the Peru Basin^36–38,52,53^, suggesting that ligands are more abundant in areas where there is also higher surface water primary productivity and POC flux^54^ (Fig. 3). The higher the POC flux, the more organic matter is usually degraded in the sediment^37^, leading to higher [dCu] and [L] in the (surface) pore waters. This assumption is in large supported by Fig. 3 which shows the relationship between [L] and POC flux and there is a trend towards higher [L] with increasing POC flux. The [L] difference between shallow and deeper pore water is, however, large in the CCZ German area, obscuring a strong and clear trend. Nevertheless, [L] is generally elevated in the Peru Basin pore waters, where there is also the highest POC flux (Fig. 3).

**Figure 5:**
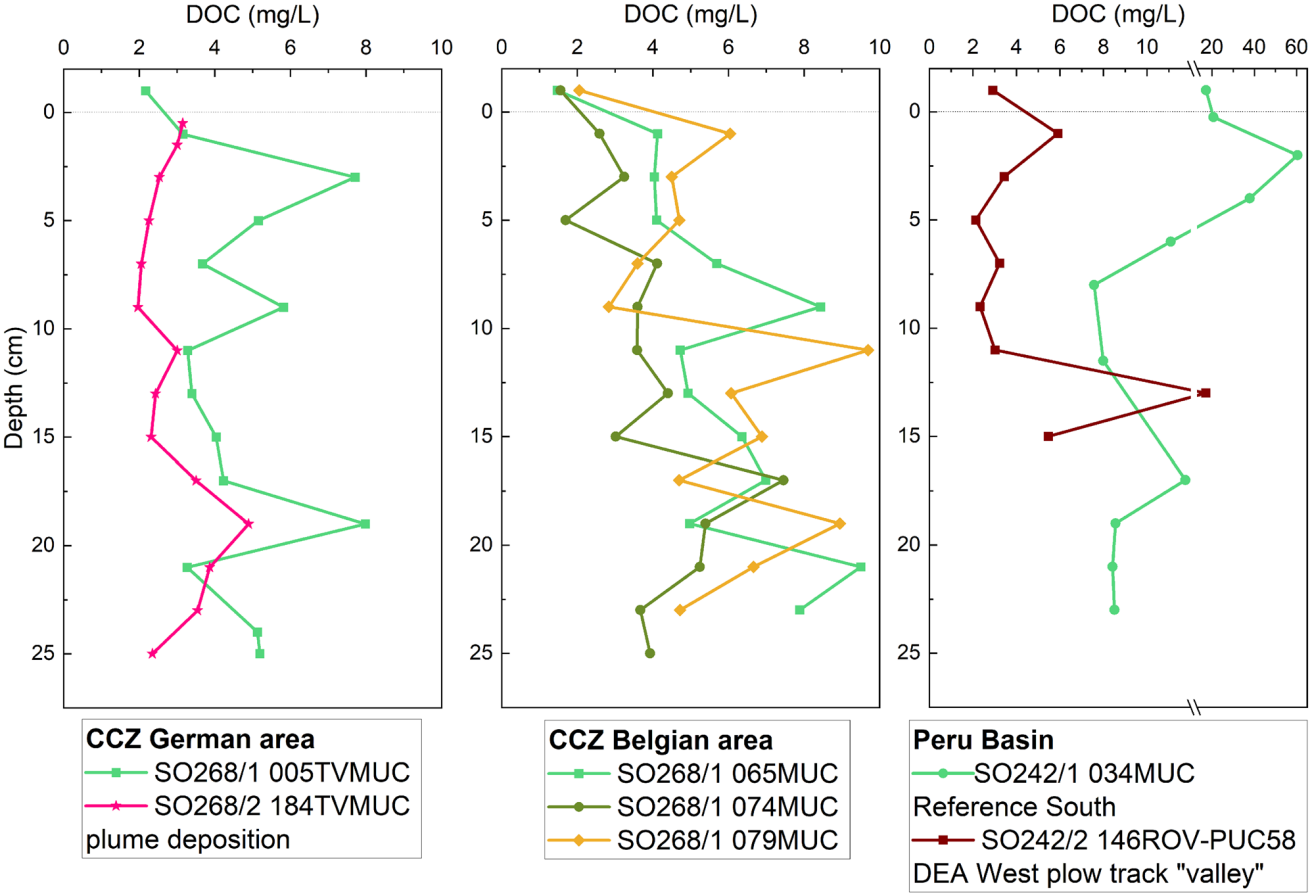
DOC concentration depth profiles in selected cores of the study sites. The uppermost (− 1 cm) value shows the bottom water. Note: Different concentration axes scales. DOC measurements were performed on a different core than ligand analyses. Data from the Peru Basin is from^60,61^.

Dissolved organic matter (DOM) released during particulate organic matter degradation might be a potential source of organic L in deep-sea pore waters and specifically refractory DOM has been previously suggested as a strong metal binding L^55^. For instance, Fe-binding L derived from refractory dissolved organic carbon (DOC; fraction of the DOM pool) have been shown to be an important L source in the North Atlantic deep ocean with stronger Fe complexation in relation to labile DOC^56^. In some marine environments, DOC shows a positive correlation with Cu-binding L, as many organic molecules that act as L are part of the bulk DOC pool^57,58^. In these regions, DOC can be a good predictor for L. There is, however, no correlation between DOC and [L] in our study, which could be because L are only a small fraction of the DOC pool^59^. However, undisturbed pore-water DOC concentrations at our study sites, even though variable with depth, were on average higher in the undisturbed pore water than in the MUC bottom water (Fig. 5). This is in general the same trend as observed for L (Fig. 2).

Labile and refractory DOC in general seem to be, however, comprised of weaker metal-binding L than the actively produced L from plankton because metals such as Fe that are bound to DOC are more bioavailable^56^. The labile DOC concentration commonly decreases rapidly throughout the water column while refractory DOC concentrations remain constant^56^, hence in the deep sea, even though not assessed in our current study, refractory DOC might be an important part of Cu-binding L. This idea is supported by the fact that our undisturbed pore-water Log*K’*_*CuL, Cu2*+_ (11.77–12.75) were lower than LogK_1_ (12.5–16.5^3,28,51^) obtained for most surface seawaters, where L are more likely actively produced by organisms. Ligand production from plankton and other organisms has been suggested to be strongest in the euphotic zone^62^ where primary production takes place. Thiols and other reduced sulfur substances, important L groups in sulfur-rich environments such as sulfidic sediments^12^ or hydrothermal vents^33^, have also been suggested to be sourced from shallow lagoon sediments^63^. Yet, it is unclear if thiols might be important L in the oxic and suboxic deep-sea pore waters presented here and too little is known about active or passive L production in the deep sea, which should be further studied. All these molecules mentioned above can act as L but since voltammetry does not give any information of the chemical structure of L, a clear determination of their sources is impossible. Future studies have to start characterizing L beyond Log*K’*_*CuL, Cu2*+_ values to clarify this gap of knowledge.

Copper was almost exclusively complexed by organic L in shallow (< 10 cm) and deeper (> 10 cm) pore waters indicated by [L] > [dCu] (Fig. 4; 14 of 16 samples that could be fitted). Comparing [L] and [dCu] (Fig. 4, Table 2), [L] was in the same range of [dCu] in the Peru Basin undisturbed pore water. Even though [L] was high, there was no large excess because [dCu] was also comparatively high at this site. There was, however, excess [L] at depths > 10 cm (Figs. 2 and 4). In surface pore waters (< 10 cm), [L] was in excess over [dCu] by a factor of 3–5 in all undisturbed cores from the German area and by a factor of 5 in one core from the Belgian area (079MUC). The other core from the Belgian area (074MUC) had [dCu] in excess over [L]. Pore waters extracted from > 10 cm in the CCZ showed a factor 1–3 excess of [L] over [dCu]. Overall, ca. 60% of all samples had a higher [L] than [dCu] and ca. 80% of samples that could be fitted with either ProMCC or Excel; this was especially the case for the surface pore waters < 10 cm (Fig. 4) and showed that [L] can buffer potential Cu^2+^ toxicity.

**Figure 6:**
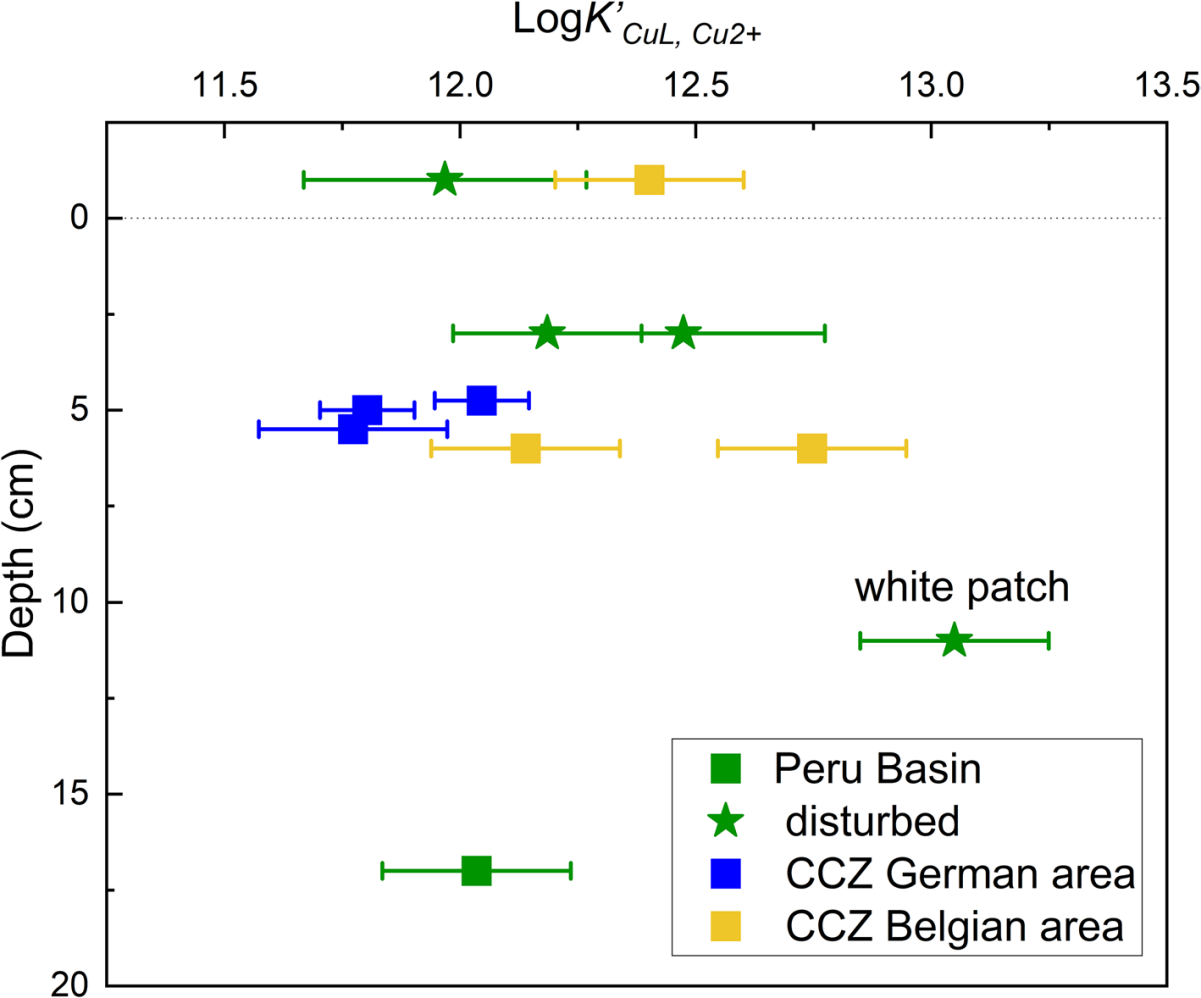
Log*K’*_*CuL, Cu2*+_ values vs. depth for all samples that could be fitted with ProMCC: 5/10 Peru Basin, 3/18 CCZ German area, 3/9 CCZ Belgian area. For details see Table 2. Samples from each study site are grouped. The uppermost (-1 cm) value shows the bottom water.

While > 99% Cu was still complexed by organic L in the deeper pore waters in the one undisturbed sample that could be fitted with ProMCC, the ratio of [L]:[dCu] decreased from 3.1 in the surface pore water < 10 cm to 1.9 in pore waters > 10 cm of undisturbed sites. Especially in the CCZ, [L] decreased with depth (Fig. 2). Organic matter degradation predominantly occurs in the surface sediment and the labile organic matter content decreases within 10 cm or 20 cm in the Peru Basin^36^ and CCZ^37^, respectively, which explains the greater availability of organic matter including organic ligands in the surface pore waters < 10 cm. The overall [L] decrease with depth in the sediments of the undisturbed sites (Figs. 2 and 4) suggests that larger amounts of labile L material are available at the sediment surface and less in deeper layers. Unfortunately, the Log*K’*_*CuL, Cu2*+_ values of shallow and deeper pore water could not be compared to assess ligand strength because the deeper pore-water samples could only be fitted for the Peru Basin samples, where one disturbed site showed higher Log*K’*_*CuL, Cu2*+_ (13.05 ± 0.2) than the one undisturbed site (12.04 ± 0.2) (Fig. 6). Considering possible active ligand production, it is also likely that fewer organisms live and therefore excrete less Cu complexing L at greater depth within the sediment. The upper 10–20 cm are the most biologically active, bioturbated and labile organic matter-rich layers of deep-sea sediments^35,36^, and it is thus not surprising that [L] is higher. Future studies should analyze pore waters from deeper layers (several meters) than was possible here to see if this downward trend of decreasing [L] is confirmed. Additionally, deeper samples from suboxic pore waters of the study sites could result in interesting insights and should also be studied in the future. In suboxic pore waters, competition from other metals such as Fe or Ni, released during reductive dissolution of Mn oxide and Fe oxyhydroxide, for the metal-binding ligand could lead to a smaller fraction of [CuL]. For the 6 undisturbed pore water samples that could be fitted in ProMCC, the toxic [Cu^2+^] were low. In the CCZ, pore waters < 10 cm had [Cu^2+^] between 4.00 and 5.68 pM in the German area at undisturbed sites. In the Belgian area, [Cu^2+^] was between 2.03 and 323 pM, the 323 pM for 74MUC being an exception (Table 2). We recognize that this value lies above the toxicity threshold for some marine organisms, but since this area is undisturbed and the value is an exception from the rest of the data, further studies in this area are needed to better evaluate the environmental risk of this area. The CCZ deeper pore waters could not be fitted with ProMCC. Overall, [Cu^2+^] was generally lower in the Peru Basin pore waters than in the CCZ pore waters (Table 2).

Compared to coastal pore waters from Chesapeake Bay^12^, the only other studied site for pore-water Cu-binding L we are aware of, [dCu] was elevated in our deep-sea pore waters, while [L] was in the same range or lower (0.1–24.3 nM [dCu] and 170–18,920 nM [L] at Chesapeake Bay compared to 3–96 nM [dCu] and 6–313 nM [L] in the deep sea). [L] were highest at the muddy, sulfidic station and lowest at a slope station at Chesapeake Bay^12^. The conditional stability constants were considerably higher in the deep sea with Log*K’*_CuL, Cu2+_ (11.77–12.75) compared to those of Chesapeake Bay (7.2–11 and 6.2–10 for LogK_1_ and LogK_2_, respectively)^12^. The methods used to determine L in the coastal pore waters was, however, ASV, which is commonly known to lead to lower Log*K* values compared to CLE-CSV for seawater samples. Hence, the values of the two studies are difficult to directly compare^28,64^. Potentially, stronger ligands are present in deep-sea pore waters compared to shallow, more terrestrially influenced, organic-rich coastal waters because they are more refractory, i.e. more difficult to degrade by microorganisms^65^. However, more research is needed to corroborate this suggestion. Pore-water L analyses currently suffer from small sample volumes that are often not sufficient to perform ligand titrations with sufficient titration points for good fitting procedures using ProMCC. Nevertheless, the pilot study data presented here gives a first indication that relatively strong (Log*K’*_*CuL, Cu2*+_ 11.77–13.05) Cu-binding L are abundant in deep-sea pore waters that can buffer Cu^2+^ released during mining activities and that dCu is > 99% present as CuL.

### Benthic flux of dCu and organic ligands in natural conditions

dCu is released at the sediment-water-interface during organic matter degradation^18,48,66^ and remains in solution due to strong organic L complexation^1^. The high concentration peaks of [dCu] and [L] below the sediment-water-interface (Fig. 2) suggest a benthic flux of dCu – predominantly organically complexed – and of free and uncomplexed L to the seawater, corroborating that pore waters are a source of L to the seawater, as has been previously suggested for coastal and slope sediments^12,34,63^. As many Cu-binding L are part of the DOC pool, higher DOC concentrations in the pore water compared to bottom water are indicative of a potential L flux from the sediment to the water column (Fig. 5,^34,67^). Considering the large extent of deep-sea seafloor, the contribution of [L] from pore waters might be significant on a global scale influencing the cycle and fate of Cu over large spatial scales. Pore-water derived L might also be important for other bioactive metals such as Fe and Zn, a large percentage of which are also often organically complexed in marine systems^68,69^. The abundance of CuL discovered in this study also suggest that care needs to be taken when calculating diffusive fluxes of dCu and potentially other trace metals across the sediment-water-interface. The diffusion of dCu out of the sediments is often calculated considering Cu^2+^^18,70^, but the flux could be significantly different, if Cu is > 99% complexed by organic L since CuL diffuses at a different rate than Cu^2+^^12,70^.

### Copper toxicity and changes to the organic ligand pool in a deep-sea mining scenario

The most toxic form of Cu is the free ion (Cu^2+^) together with labile, mostly inorganically complexed Cu (CuX_IN_)^59,71^ because these two Cu forms are considered the most bioavailable. Deep-sea mining might have the following impacts on Cu toxicity and the Cu cycle: (1) sudden release of dCu and L from the pore water, where [dCu] and [L] are elevated compared to seawater, (2) changes in L availability in the plume affected water column due to changed biogeochemical conditions, and (3) changes of L availability in the pore water due to removal or deposition of sediment, including changes in organic matter quality or quantity in certain sediment layers.

The excess of [L] over [dCu], especially in the undisturbed surface pore waters < 10 cm (Figs. 2 and 4), suggests that Cu released to the water column in a mining scenario is present in an organically complexed form of commonly low bioavailability^3^. The large excess of L indicates free, uncomplexed L that can also complex additional, released Cu^2+^. Toxic effects from a sudden release of Cu from pore waters to bottom seawater seem therefore negligible but no final conclusions can be drawn about the toxic or limiting effect of [Cu^2+^] on prevalent deep-sea fauna because threshold data is missing for such species and there is no [Cu^2+^] data from shortly after a disturbance. Additionally, cumulative impacts resulting from the release of higher concentrations of several metals from pore waters including e.g., Mn and Cd, should be considered, but little data on such multi-stressor effects is available so far. Thus, drawing general conclusions after studying one metal is not possible and would be misleading because toxicity assessments may vary strongly between faunal species and metals^17,24^. For instance, while hydrostatic pressure was shown to increase toxic effects of Cu in a shallow-water shrimp, this was not the case for Cd^17^. Additionally, it is difficult to give concrete recommendations for threshold values because impacts can be diverse depending on species and life stage^24^; macro- or megafauna and adults might have a higher resistance against Cu than microbes and larvae. Especially microbial communities have gained little attention regarding ecotoxicological effects of metals in the deep-sea plains^72,73^ despite them being an important part of the basis of the deep-sea biomass production and nutrient regeneration in polymetallic nodule fields^73^. Studies with hydrothermal vent microbes revealed that these organisms can tolerate [dCu] up to 10 µM by actively producing ligands^9^. Nevertheless, care needs to be taken when extrapolating these findings to deep-sea abyssal plains, where microbes are never naturally exposed to such high [dCu] (and much lower [Cu^2+^]) but have to cope with multiple stressors that can affect the organism’s Cu^2+^ tolerance level. Moreover, even though non-organically-complexed dissolved metals are more likely to be toxic than particulates^74^, the impact depends on the species and not only dCu and Cu^2+^ should be studied to assess toxicity, but also colloidal and particulate Cu. Filter and benthic deposit feeders, for example, might be more impacted by ingested (nano)particulates compared to other non-filter feeding organisms^75,76^. While nanoparticulate Cu is taken up more slowly than Cu^2+^, it has also been found to be excreted more slowly than Cu^2+^, remaining in the organisms for a longer time^77^. Thus, cumulative impacts of dissolved phases with a focus on free species and (nano)particulate phases should be studied in the future together with impacts of simultaneously increased levels of various bioactive metals, to improve current risk assessments of marine environments^24^.

It is also worth noting that bioavailability and toxicity of Cu are considerably decreased by higher pH and higher DOC concentrations^78^. As pH decreases, H^+^ ions compete with Cu^2+^ for DOC binding sites and therefore less Cu^2+^ is complexed by DOC^78^. Copper complexed by DOC is assumed to be largely non-bioavailable^78^. Ex-situ experiments with sediment cores simulating a sediment disturbance discovered a pH decrease in the particle suspension post-impact^79^, which suggests less Cu^2+^ might be complexed by DOC during deep-sea mining in the sediment plume. Hence, a reduction in the available Cu-binding sites of organic L could increase the level of Cu^2+^ and therefore toxicity in affected deep-sea habitats. The number of DOC binding sites for Cu^2+^ might be further reduced by other cationic metals (e.g., Ni, Zn) released with the sediment plume and competing for binding sites with Cu. On the other hand, L might be supplied in the sediment suspension. Ex-situ disturbance experiments with cores previously showed that fresh organic matter rich in amino acids is found in the suspension when the sediment is disturbed^79^. As some amino acids also act as organic L for Cu, the [L] in the bottom water could also increase post-disturbance for a short time. All in all, it remains unclear, whether the dominating process will be an increase of [L] and thereby more binding capacity or reduced binding capacity due to a lower pH and competition with other metals. It is, however, unequivocal that the biogeochemical equilibrium will be changed due to the impact. Besides organic complexation, it has been shown that scavenging onto negatively charged Mn-oxide surfaces of particles in the sediment plume is one of the main removal mechanisms of cationic heavy metals such as Cu^2+^ in the water column^21^, which could further decrease [Cu^2+^] and thereby toxicity. Such Mn-oxide particles are usually more abundant in the plume associated with the sediment disturbance and resuspension processes than in the undisturbed water column. However, depending on the size of the Mn-oxide particles, their residence time in the water column can vary and Mn-oxide nanoparticles and colloids (NPCs) can act as CuX_IN_ with higher bioavailability than organically complexed Cu. Mn-oxide NPCs therefore do most likely not decrease Cu bioavailability, only Mn-oxide particles in the µm range will. Since the bottom water [dCu] was found to be 6.8 nM 5 weeks post-impact (184TVMUC), dCu seems to have been largely removed from the water column during plume settling. More research such as a time series post-disturbance is needed to understand the predominant mechanism controlling Cu speciation and residence times in the bottom water during deep-sea mining plumes.

Post-disturbance, it is unclear how long the biogeochemical equilibration of the Cu cycle will take. At the 26-year-old disturbed sites in the Peru Basin, shallow pore-water [L] < 10 cm and deeper pore-water [L] was in the same range as the undisturbed site (Table 2). There was no clear trend of excess [L] in the shallow pore water (< 10 cm) but the deeper pore waters > 10 cm that could be fitted had excess [L] (Figs. 2 and 4). In the Peru Basin, [L] in the surface pore waters < 10 cm is lower than in the deeper layers at the disturbed sites, contrary to the profile shapes of all undisturbed sites in the CCZ, but the Reference South undisturbed site in the Peru Basin shows a similar trend (Fig. 2). At the seafloor, a disturbance can lead to mixing or turn-over of surface sediments, which brings labile organic matter from the surface into deeper layers. The introduction of labile organic matter into deeper layers could provide additional organic ligands to the deeper pore water. This process could explain why [L] is in excess of [dCu] at DEA South “white patch” in the Peru Basin > 10 cm (Fig. 2). But if the [L] increase at depth is a disturbance feature or within the natural variability is unclear and more undisturbed sites from the Peru Basin would need to be assessed. Interestingly, the “white patch” site > 10 cm also shows the highest Log*K’*_*CuL, Cu2*+_ value (13.05) overall (Fig. 6), suggesting slightly stronger ligands at this site. For all other disturbed sites that could be fitted with ProMCC, there is no discernable difference in L strength because the Peru Basin disturbed sites fell into the same range as the undisturbed sites (Fig. 6).

DOC concentrations in the disturbed cores are lower than in the undisturbed cores from the same areas (Fig. 5), suggesting that cycling of organic matter is impacted, which might also impact L availability. But a larger data set should be studied to confirm these trends and more Cu speciation data from disturbed sites is needed as well. A time series would also need to be conducted for impacted pore waters to assess short-term and long-term changes.

Unfortunately, the plume deposition site in the CCZ German area could not be fitted for the surface pore water, so the impact of resettled material on L cannot be examined. [L] > 10 cm at the plume deposition site was in the same range as the undisturbed sites (Table 2). None of the samples from the CCZ from > 10 cm could be fitted with ProMCC but regardless of their [Cu^2+^], mining is expected to impact ca. the upper 12 cm^80^, so that most pore water below would not be directly released in a disturbance event.

## Conclusions

Our data indicated that relatively strong (Log*K’*_*CuL, Cu2*+_ 11.77–13.05) Cu-binding organic L were abundant with up to 313 nM in deep-sea pore waters, particularly in shallow pore waters < 10 cm, of the CCZ and Peru Basin in both undisturbed and disturbed sites, and that dCu was > 99% complexed by organic L for 8 of 9 pore water samples. Hence, dCu released from the pore water to the bottom water during a disturbance of the surface sediment layer, such as deep-sea mining, can be assumed to be predominantly present in a complexed form which is less bioavailable than Cu^2+^ or CuX_IN_. This assumption is further supported by the fact that [Cu^2+^] were except in one sample < 6 pM, both at 26-year-old disturbed and undisturbed sites. Moreover, the excess of L found especially in shallow pore waters implied that even with a Cu release through mining activities, Cu^2+^ likely remains beneath toxic thresholds because there was excess L that can buffer toxicity. With this, the potential toxic risk of dCu release from the pore waters due to deep-sea mining activities can be considered negligible. More research is necessary to assess cumulative effects of different size fractions of Cu, the co-release of several metals, and variations in pH on various deep-sea species to evaluate the risk of mining activities on prevalent biota more accurately.

## Material and methods

### Pore-water sampling

Samples were taken during RV SONNE cruises SO242 (2015)^40^, SO262 (2018), and SO268 (2019). During SO242, samples were collected with push cores (PUC) using the ROV KIEL 6000 from GEOMAR, while samples during SO262 and SO268 were taken with a multicorer (MUC) or video-guided MUC (TVMUC). Pore water for low resolution dCu and Cu speciation analyses was extracted using rhizons (pore size 0.12–0.18 µm, from Rhizosphere Research Products) that were inserted into predrilled holes in the plastic MUC or ROV-PUC liner in the cold room of RV SONNE at ca. 4–7 °C. Since little information exists on the reliability (i.e. recovery, contamination) of rhizons for trace metal sampling, we tested if the rhizons reliably sample Cu, which our tests confirmed, see Supplementary 1 for details. Rhizons used on SO242 were deionized ultrapure water (> 18.2 MΩ/cm; DI) or acid-cleaned. Rhizons used on SO262 were pre-cleaned with 0.1 M suprapure HCl and DI prior to and between sampling. Rhizons used on SO268 were cleaned with DI before the first use and with 0.1 M suprapure HCl and DI between uses. Syringes were always cleaned with 0.1 M suprapure HCl and DI. The first mL of sample was used to rinse the rhizon and syringe with sample and was subsequently discarded. Usually pore water from three rhizons, corresponding to three depths, was pooled to get sufficient volume (~ 100 mL) for the Cu speciation analyses and low resolution dCu analyses. In the CCZ, where the MUCs are oxic, the differentiation was made between bottom water, surface sediment (ca. upper 10 cm) and deeper layers 10–30 cm. In the Peru Basin, where MUCs are suboxic below ca. 15–20 cm^18,36^, sampling was divided into bottom water, oxic surface pore water (ca. 5 cm) and suboxic pore water based on the absence of the Mn-oxide rich dark brown top layer during sampling. The pore waters sampled at ca. 10–20 cm depth are, however, predominantly oxic as determined in later oxygen and trace metal analyses^18^. An aliquot of each of the pooled samples was used to measure pH (total scale) and salinity with a WTW® multimeter. Pore water was stored in pre-cleaned fluorinated Nalgene bottles (or low-density polyethylene bottles (LDPE) bottles for SO242) and frozen at -20 °C directly after sampling and kept frozen until analysis. Bottles were pre-cleaned using organic detergent (neodisher® LaboClean A8; only for SO262 and SO268) in a dishwasher, 2% HNO_3_ and 0.2% HF acid mix (2 days at 45 °C), and DI.

Pore water for the quantification of dCu at higher (2 cm) resolution and DOC analyses was extracted by centrifuging subsampled sediment from other MUC liners than used for ligand sampling, but from the same MUC deployment, for 40 min at ca. 2061–2465×*g* relative centrifugal force. The supernatant was filtered using syringes with 0.2 µm polycarbonate (SO262) or polyethersulfone (PES; SO268) filters that were pre-cleaned with 0.1 M suprapure HCl and DI. dCu samples at higher resolution were collected in pre-cleaned LDPE bottles, cleaned according to the protocol above (with an additional 1 M suprapure HCl (2 days at 45 °C) step for SO268), while samples for DOC analyses were collected in high-density PE bottles cleaned with organic detergent (neodisher® LaboClean A8) in a dishwasher and 1 M suprapure HCl (2 days at 45 °C). Samples for dCu at higher resolution and DOC were subsequently acidified to pH 1.8–2 with ultrapure HCl.

### dCu analyses with ICP-MS

All high resolution dCu samples as well as low resolution dCu samples of SO262 and SO268 were measured using an Inductively Coupled Plasma—Mass Spectrometer (ICP-MS) Nexion 350x (PerkinElmer) coupled to an *apex* Q (ESI) for improved sensitivity and reduced background at Jacobs University Bremen (Germany). To reduce polyatomic ion interferences, Cu was measured in KED (kinetic energy discrimination) mode. Performance was checked by repeated measurements of the estuarine reference material SLEW-3 (National Research Council Canada) which has a [dCu] of ~ 24.39 ± 1.89 nM. Our data are in very good agreement with the SLEW-3 reference value (25.3 ± 3.2 nM, n = 7 ICP-MS run averages). Rhizon blanks taken from DI on SO262 and SO268 were below the detection limit of the instrument (detection limit = 0.66–4.00 nM, n = 8 ICP-MS runs, detection limit determined for each run). Samples for which ICP-MS [dCu] were below the limit of quantification (LOQ, 10 × standard deviation of blank) of ca. 11 nM were remeasured with voltammetry, which has a lower detection limit (see section below). Low resolution dCu samples from SO242 (Peru Basin) were also measured using voltammetric techniques (see section below). Based on the comparison of five samples that were between 7–10 nM for ICP-MS measurements and were repeated with the voltammetric method, concentrations were on average 10% lower in voltammetric measurements than in the ICP-MS measurements, with a range of 25% lower to 12% higher for the voltammetric measurement. Considering the different methods and that the concentration range is low for the ICP-MS, the agreement was considered acceptable. The remeasured data was significantly higher for samples SO262 063MUC bw, SO268 65MUC 15, 18, 22 cm and SO268 79MUC 17, 20, 23 cm, which might be associated with contamination during sample handling for voltammetric measurements so we used the ICP-MS data for these samples.

### Sample preparations, total dCu analysis, and CLE–AdCSV titration

Total dCu and Cu speciation analysis was carried out using a 757 VA Computrace stand (Metrohm) coupled with VA Computrace Software 2.0 (Metrohm) at Jacobs University Bremen. The three-electrode configuration is composed of a hanging mercury drop electrode (HMDE) as the working electrode, an Ag/AgCl reference electrode, and a glassy carbon counter electrode. All reagents and stock solutions were prepared according to the methods described in^32^.

In the case of total dCu analysis, 10–20 mL of samples were pipetted and acidified to pH 2 with concentrated suprapure HCl (30%, Merck). Samples were then UV-digested following the protocol of^32^. Using CLE-AdCSV^32^ (Supplementary 2) coupled with a Cu standard addition method (0 nM, 15 nM, 30 nM, and 60 nM except for NASS-6 for which 0 nM, 5 nM, 10 nM and 20 nM) dCu was determined in the samples. Before the measurement of each sample, the voltammetric vial was preconditioned with suprapure NaCl (32 ppt, Merck) and borate buffer for at least 24 h to ensure peak height stability. Measurements were done in triplicates using differential pulse mode (DP), a mercury drop size of 4 mm, and a scan from − 0.05 V to − 0.8 V. Other voltammteric parameters used can be found in the literature^10,32^ and are detailed in Supplementary 2. Peak height was chosen as the characteristic signal value. Data quality was checked by measuring blanks and a reference material (NASS-6; National Research Council Canada) after the same sample treatment. Blanks yielded [dCu] of 0.22 ± 0.10 nM and NASS-6 gave results (3.5 ± 0.1 nM, n = 3) within the range of the consensus value (3.9 ± 0.4 nM). The detection limit of our method was 0.29 nM (3 × the standard deviation of the blank measurements).

Cu speciation in the samples was determined with CLE-AdCSV^81^ using the same instrumental settings as for the dCu analysis (Supplementary 2). Before each titration, 5 mL aliquots of each sample were used for conductivity measurements (WTW) to determine the concentration of suprapure NaCl solution needed for the dilution of samples with restricted volumes (< 90 mL). Samples were diluted with NaCl solution (32 ppt NaCl; similar to the salinity of original samples) using a dilution factor of 1–3 for Peru Basin samples and 10 for CCZ samples. Acid cleaned and pre-conditioned (NaCl of 32 ppt, borate buffer, and Cu in a titration range of 0–300 nM) polytetrafluoroethylene (PTFE) vials were used for the Cu titrations. 10 mL (final volume after proper dilution) of each sample was then pipetted into 9–12 vials. Subsequently, borate buffer was added into each vial to maintain the analytical pH at around 8.1. The vials were then spiked with Cu in increasing concentrations, similar to the preconditioning step. After adding Cu, the aliquots were left for 30 min before the salicylaldoxime (SA) was added to a final concentration of 5 μM. This titration window of 5 μM SA was chosen to quantify both weak and strong ligands in the pore-water samples. There was not sufficient sample volume to test multiple detection windows and thus 5 µM SA was chosen since lower detection windows (ca. < 2.5 µM SA) do not resolve and/or underestimate strong L concentrations^82^. Afterward, the aliquots were left in the PTFE vials for at least 16 h to let the complexation reactions between Cu and SA reach equilibrium.

For each titration, the concentration of complexing ligands ([L]) and corresponding conditional stability constants (Log*K’*_*CuL, Cu2*+_) were determined using the fitting software named ProMCC^50^. [dCu] from the low-resolution, pooled Cu profiles were used, so the dCu data used for fitting is from exactly the same sample bottle as the L data. Data was fitted using both the one-ligand and two-ligand complete complexation-fitting model^50^. The model with the best fit and thus the least fitting error was used for data interpretation, which was the one-ligand model. Side reaction coefficients and stability constants of SA of each sample were provided by van den Berg’s ion-pairing model for seawater (EXCEL worksheet—ion-pairing model for seawater, written by C.M.G. van den Berg in 2014) using sample specific salinity values measured in the lab, a SA concentration of 5 µM, an ambient temperature of 21 °C, and a pH of 8.1. Details from ProMCC fitting are provided in the speciation table in Supplementary 3. Samples that could not be fitted with ProMCC because of too few titration points below and within the linear section of the titration curve were fitted with Excel using the least squares method (linest function). The linest function is commonly used in chemistry to find unknown concentrations from a calibration curve. For our samples, the titration curve is only linear when the natural ligands are saturated with Cu and added Cu solely binds to the excess of SA in the sample because CuSA is electrolabile and hence responsible for the signal. The more Cu is added, the more Cu binds to SA (because the natural ligand is already saturated), and the higher the signal. Hence, where the CuSA titration curve gets linear CuL = CuSA. Consequently, the total ligand concentration ([L_T_]) of the sample can be calculated via the X-intercept. For examples of titrations that could or could not be fitted with ProMCC see Supplementary 4.

The main improvement for future studies of pore water ligands should be the development of voltammetric methods that need smaller sample volumes, to test multiple detection windows and obtain titrations with a sufficient resolution for subsequent data fitting. Too much pooling of different cores or over extensive depth ranges to get larger pore water volumes than for this study will mask the small-scale heterogeneity of ligand distributions and would not be a recommended alternative option.

### Dissolved organic carbon (DOC)

DOC samples from SO268 were analyzed at Jacobs University Bremen with a high temperature combustion method using a multi N/C 2100S (Analytik Jena). Sample vials were pre-combusted overnight at 500 °C prior to use and samples were diluted with DI if insufficient volume was available. Inorganic carbon was removed from the sample through acidification with HCl (~ pH 2) at the time of sampling and a five-minute purge with synthetic air. The measured DOC concentration was an average from 2–3 measurements depending on the variance of the measurement. All samples were additionally analyzed in duplicate. To check for accuracy, the deep-sea reference (DSR) material batch 16, lot# 11–16^83^ was measured (0.55 ± 0.05 mg/L, n = 10) and agreed well with the reference value of 0.53–0.54 mg/L. Method blanks of DI, processed like samples on the ship, had a DOC concentration of 1.44 ± 0.62 mg/L (n = 4) for 0.2 µm PES filter. As this method blank concentration was quite variable, close to the concentration of some measured samples, and we are not sure how much DOC comes from the filtration process/materials, this blank was not subtracted from the final DOC results. A slight overestimation of the sample DOC is thus possible. Finally, the DOC was stated as the average of duplicate measurements.

## Supplementary Information

Supplementary Information.

## Data Availability

Data are available at PANGAEA: https://doi.pangaea.de/10.1594/PANGAEA.932940.
