# Supplementary material for: Copper-binding ligands in deep-sea pore waters of the Pacific Ocean and potential impacts of polymetallic nodule mining on the copper cycle
Source: Sci Rep. 2021 Sep 16;11:18425. doi: 10.1038/s41598-021-97813-3 (PMC8446087; doi:10.1038/s41598-021-97813-3)

Supplementary

Copper-binding ligands in deep-sea pore waters of the equatorial Pacific Ocean and potential impacts of polymetallic nodule mining on the copper cycle

Sophie A. L. Paul[1,2*], Rebecca Zitoun[3], Ann Noowong[1], Mythili Manirajah [1], Andrea Koschinsky[1]

[1] Department of Physics and Earth Sciences, Jacobs University Bremen, Campus Ring 1, 28759 Bremen, Germany
[2] GEOMAR Helmholtz Centre for Ocean Research Kiel, Wischhofstr. 1-3, 24148 Kiel, Germany
[3] Department of Ocean Systems (OCS), Utrecht University, Royal Netherlands Institute for Sea Research (NIOZ), 1797 SH, 't Horntje, The Netherlands
*Corresponding author

Supplementary 1 Rhizon testing

Little information exists on the reliability (i.e. recovery, contamination) of rhizons for trace metal sampling (e.g., rare earths (Abbott et al., 2015)). However, the data that is available suggests that rhizons do not quantitatively sample metals from solution or that a significant blank is introduced. Therefore, we tested two new rhizons, cleaned with DI at the beginning of the experiment and cleaned with 0.1 M HCl suprapure between sampling and discarding ca. 1 mL sampled solution first, mimicking the procedure at sea. After each cleaning, a DI Blank was taken through the rhizons. With rhizon #1, we sampled a DI solution spiked with 79 nM Cu (prepared from a single element standard) and with rhizon #2 we sampled a 550 mM NaCl-DI solution (NaCl suprapure; Merck; approximately seawater salinity) spiked with 79 nM Cu. Blanks and both test sets were measured with ICP-MS (for details also see Material and methods – dCu analyses with ICP-MS). The concentration of 79 nM was chosen to reliably measure it with the ICP-MS at 80x dilution and not have issues with the detection limit. An aliquot of the Cu-spiked solutions was kept at the beginning of the experiment as a reference for the original solution. At the end, an aliquot from the final solution was kept again to account for possible wall adsorption throughout the time of the experiment or any other impacts on the Cu spiked solutions.

Results showed that blanks (0.5 M HNO_3) were below the detection limit for Cu (0.4 nM, n=1 ICP-MS run) except the second rhizon #2 blank after HCl cleaning. Recovery of the original 79 nM solution at the end was 100-104% (Table S1). Recovery of the spiked DI solution was only 34-53%, while recovery of the NaCl-solution was 103% (Table S1). The quantitative NaCl recovery of 103% suggests that rhizons can indeed be used for pore-water Cu sampling in seawater.

Table S1: Results from rhizon testing incl. Blanks, 79 nM Cu spiked DI, and 79 nM Cu spiked NaCl-DI solution. LOD: limit of detection (0.4 nM).

Sample ID	[nM]	% Recovery
Blank rhizon #1 new DI clean	< LOD	
Blank rhizon #1 (reused) HCl clean	< LOD	
Blank rhizon #2 new DI clean	< LOD	
Blank rhizon #2 (reused) HCl clean	1.0	
79 nM Cu DI original beginning	79	
79 nM Cu DI rhizon #1 new DI clean	42	53
79 nM Cu DI rhizon #1 HCl clean	27	34
79 nM Cu DI original end	79	100
79 nM Cu NaCl original beginning	79	
79 nM Cu NaCl rhizon #2 new DI clean	81	103
79 nM Cu NaCl rhizon #2 HCl clean	81	103
79 nM Cu NaCl original end	82	104

Reference:

Abbott, A.N., Haley, B.A., McManus, J. & Reimers, C.E., 2015. The sedimentary flux of dissolved rare earth elements to the ocean. Geochimica et Cosmochimica Acta 154: 186–200.

Supplementary 2 Voltammetry parameters for CLE-AdCSV used in this study

Table S2: Voltammetry parameters for CLE-AdCSV used in this study (DP Mode). All voltammetric parameters were adapted from Sander et al. (2007).

Parameter categories	Setting parameter	Setting value
N_2 gas	Pressure (bar)	1.0 - 1.2
Voltammetric setting	Initial purge time (s)	120
Pre-treatment setting	Deposition potential (V)	-0.0499
	Deposition time (s)	60
	Equilibration time (s)	5
Sweep setting	Start potential (V)	-0.0499
	End potential (V)	-0.6
	Pulse amplitude (V)	0.07004
	Pulse time (s)	0.025
	Voltage step (V)	0.005035
	Voltage step time (s)	0.3
	Sweep rate (V/s)	0.0168

Reference:

Sander, S. G., Koschinsky, A., Massoth, G., Stott, M. & Hunter, K. A., 2007. Organic complexation of copper in deep-sea hydrothermal vent systems. Environmental Chemistry 4: 81–89.

Supplementary 3 Speciation table from ProMCC fitting

The resulting detection window of the method (D_{SA}) was ~4.3 logD_{SA}, calculated as the sum of the product of [SA] and K^{cond}_{CuSA} for CuSA and $Cu(SA)_2$ (Apte et al., 1988; Laglera et al., 2015). Complexation capacities (log$\alpha_{CuL,\ Cu^{2+}}$, with $\alpha_{Cu^{2+}L}$calculated as the product of [L´] and $K^{cond}_{CuL,\ Cu^{2+}}$; [L']: concentration of non-bound ligands by Cu; Gledhill and Gerringa, 2017) of the estimated Cu-binding ligands fell within one order of magnitude of D_{SA} indicating that Cu speciation parameters were reliably determined, except for SO242/2 219ROV-PUC28 – 10,11,12 cm.

References:

Apte, S.C., Gardner, M.J. & Ravenscroft, J.E., 1988. An evaluation of voltammetric titration procedures for the determination of trace metal complexation in natural waters by use of computers simulation. Analytica Chimica Acta, 212: 1-21.

Laglera, L.M. & Filella, M., 2015. The relevance of ligand exchange kinetics in the measurement of iron speciation by CLE–AdCSV in seawater. Marine Chemistry, 173: 100-113.

Gledhill, M., & Gerringa, L. J. (2017). The effect of metal concentration on the parameters derived from complexometric titrations of trace elements in seawater—A model study. Frontiers in Marine Science, 4: 254.

Table S3: Side reaction coefficients and stability constants of each sample. Side reaction coefficients and stability constants of each samples were obtained with the van den Berg's ion-pairing model for seawater (http://www.liv.ac.uk/%7Esn35/Documents/Useful_links_html; van den Berg, 2014) using sample specific [SA] and salinity values, as well as an ambient temperature of 21°C and a pH of 8.1. Salinity values measured in each sample in the lab were used. Parameters are given for the diluted samples.

Sample ID		Salinity	aCu'	logK'CuSA	logB'CuSA2	aCuSA	logaCuSA	Cu nM	L1 [nM]	error [nM]	logK1	Cu2+ (M)	L'	DSA	logalpha
SO242/2 146ROV-Niskin	bw	35	25	9.548694824	14.9616439	40575.1743	4.608260394	10.86					-10.858	4.25	
SO242/2 146ROV-PUC28	bw	35.7	25	9.54551276	14.9570858	40207.1034	4.604302787	8.88	68.40	24.00	11.97	1.60E-13	41.763	4.24	4.59
	2, 3, 4 cm	35	25	9.548694824	14.9616439	40575.1743	4.608260394	67.89	75.40	11.30	12.47	3.00E-12	7.513	4.25	4.35
	11, 12, 13 cm	34.9	25	9.549154592	14.9623025	40628.6421	4.608832308	93.32					74.683	4.25	5.18
SO242/2 196ROV-PUC28	bw	35.7	25	9.54551276	14.9570858	40207.1034	4.604302787	22.21					-44.418	4.24	
	3, 4, 5 cm	34.8	25	9.54961568	14.962963	40682.3362	4.609405885	27.98					-41.428	4.25	
	16, 17, 18 cm	35.3	25	9.547323359	14.9596794	40416.1127	4.60655454	50.94	121.00	29.00	12.04	6.70E-13	70.063	4.25	4.88
SO242/2 219ROV-PUC28	bw	35.5	25	9.546415509	14.958379	40311.1742	4.605425448	14.47					-28.938	4.25	
	2, 3, 4 cm	34.8	25	9.54961568	14.962963	40682.3362	4.609405885	96.24	88.00	13.60	12.19	6.59E-13	-8.238	4.25	
	10, 11, 12 cm	35.3	25	9.547323359	14.9596794	40416.1127	4.60655454	44.19	147.00	15.00	13.05	1.69E-13	102.813	4.25	6.06
SO262 026MUC	bw	31	24	9.568196173	14.9895783	42907.9833	4.632538103	1.0					-0.951	4.27	
	2, 5, 8 cm	32	24	9.563094508	14.9822705	42284.678	4.626183028	3.4					-3.419	4.26	
	12, 15, 18 cm	31.9	24	9.567730333	14.988911	42850.678	4.631957698	0.6					-0.606	4.27	
	21, 25, 27 cm	32.1	24	9.562593138	14.9815523	42223.9275	4.625558627	0.5					-0.534	4.26	
SO262 063MUC	bw	31.4	24	9.56613603	14.9866273	42655.1506	4.629971479	0.4					-1.410	4.27	
	1, 5.5, 8.5 cm	32.5	24	9.560603156	14.9787018	41983.688	4.623080586	9.6	31	5.3	12.0	3.9957E-13	21.671	4.26	4.38
	12, 15, 19 cm	32.1	24	9.562593138	14.9815523	42223.9275	4.625558627	0.9					-0.905	4.26	
	23, 27, 30 cm	32.3	24	9.561595067	14.9801227	42103.2594	4.624315718	1.1					-1.136	4.26	
SO262 149MUC	bw	32.4	24	9.561098346	14.9794111	42043.3377	4.623697186	7.3					-3.622	4.26	
	1.5, 4.5, 8.5 cm	31.8	24	9.564101966	14.9837136	42407.023	4.627437786	4.5	17	4.3	11.8	5.6788E-13	12.442	4.26	3.90
	12.5, 15, 17.5 cm	32.3	24	9.561595067	14.9801227	42103.2594	4.624315718	0.6					-0.557	4.26	
	21, 25, 30 cm	31.2	24	9.5671628	14.9880981	42780.9695	4.631250622	0.7					-0.680	4.27	

Sample ID		Salinity	aCu'	logK'CuSA	logB'CuSA2	aCuSA	logaCuSA	Cu nM	L1 [nM]	error [nM]	logK1	Cu2+ (M)	L'	DSA	logalpha
SO268/1 005TVMUC	bw	33.5	25	9.555733421	14.9717263	41401.7096	4.617018275	0.6					-0.604	4.25	
	2, 6, 9 cm	35.9	25	9.544615054	14.9557999	40103.8884	4.603186483	3.9	20	5.6	11.8	4.0142E-13	16.298	4.24	3.99
	17, 20, 23 cm	35.1	25	9.548236367	14.9609872	40521.9312	4.607690135	0.8					-0.786	4.25	
SO268/2 184TVMUC	bw	35.4	25	9.546868793	14.9590283	40363.5342	4.605989186	0.7					-0.677	4.25	
	2, 5, 8 cm	35.3	25	9.547323359	14.9596794	40416.1127	4.60655454	3.4					69.371	4.25	4.09
	17, 20, 23 cm	34.8	25	9.54961568	14.962963	40682.3362	4.609405885	1.4					-1.420	4.25	
SO268/1 065MUC	bw	35.3	25	9.547323359	14.9596794	40416.1127	4.60655454	1.7					18.973	4.25	4.19
	2, 5, 8 cm	35.3	25	9.547323359	14.9596794	40416.1127	4.60655454	2.6					-2.555	4.25	
	15, 18, 22 cm	34.8	25	9.54961568	14.962963	40682.3362	4.609405885	0.3					-0.601	4.25	
SO268/1 074MUC	bw	35	25	9.548694824	14.9616439	40575.1743	4.608260394	1.2					-1.182	4.25	
	3, 5, 9 cm	35.3	25	9.547323359	14.9596794	40416.1127	4.60655454	5.8	5.0	0.9	12.7	3.2253E-11	-0.779	4.25	
	16, 19, 22 cm	34.8	25	9.54961568	14.962963	40682.3362	4.609405885	0.5					-0.483	4.25	
SO268/1 079MUC	bw	34.2	25	9.552410341	14.9669662	41009.3446	4.612882828	2.1	5.4	1.3	12.4	2.46E-13	3.335	4.25	3.93
	3, 6, 9 cm	35.1	25	9.548236367	14.9609872	40521.9312	4.607690135	1.7	7.8	2.2	12.1	2.0308E-13	6.100	4.25	3.92
	17, 20, 23 cm	34.2	25	9.552410341	14.9669662	41009.3446	4.612882828	0.6					-0.509	4.25	

Supplementary 4 “Bad” and “good” fits of titration curves

1. Upper set of four graphs: Initial ProMCC graphs (without taking points out)
2. Lower set of four graphs: ProMCC graphs with taking points out
3. For each set of four: Graph on the upper left – titration curve, graph on the upper right – Langmuir fit, graph on the lower left – Ruzic and van den Berg fit, graph on the lower right – Scatchard fit

Bad fit examples

Sample SO268_184MUC 2,8,5 cm – not enough titration points

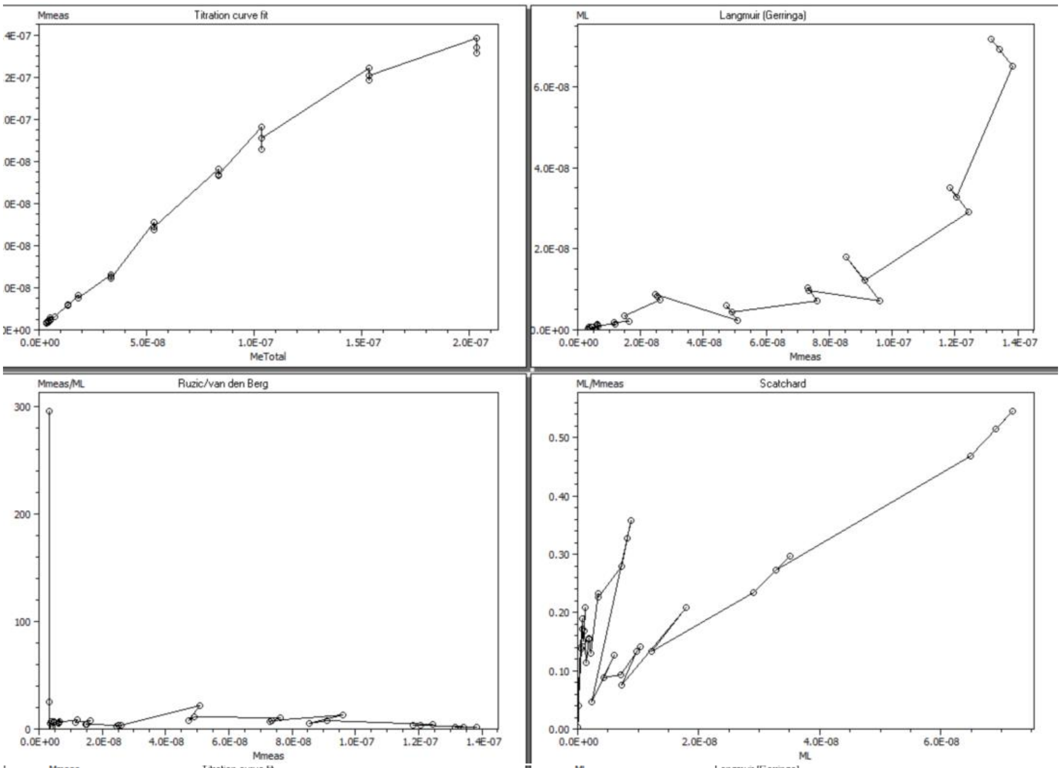

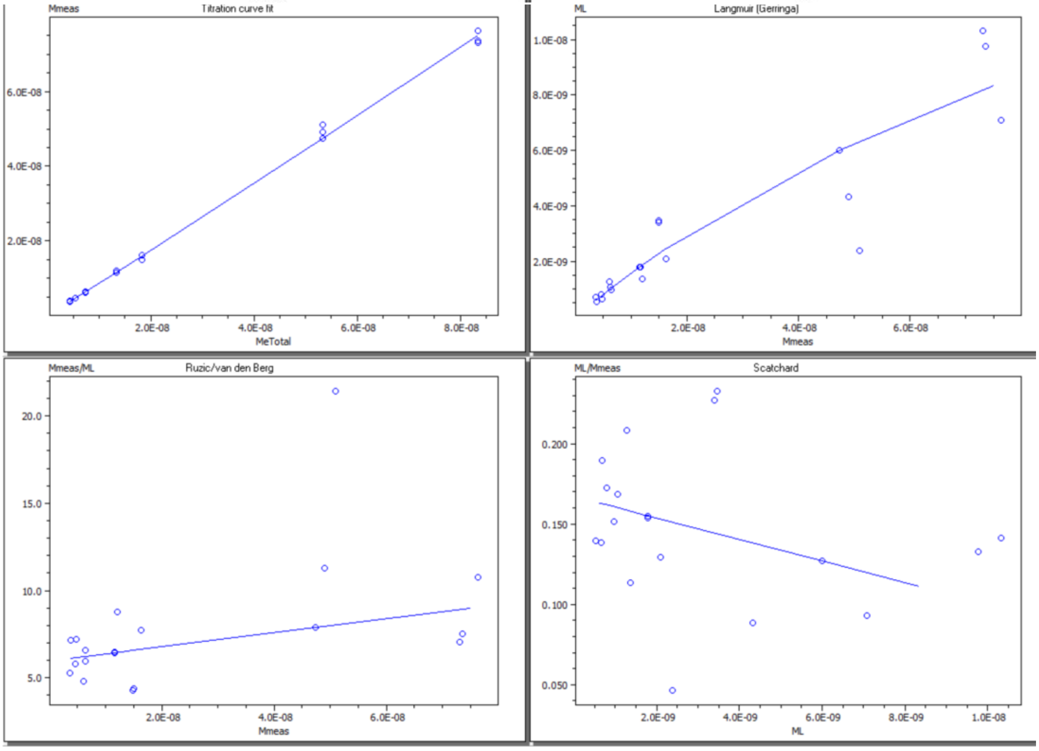

Sample SO242_146ROV28_11-13 cm – bad titration curve

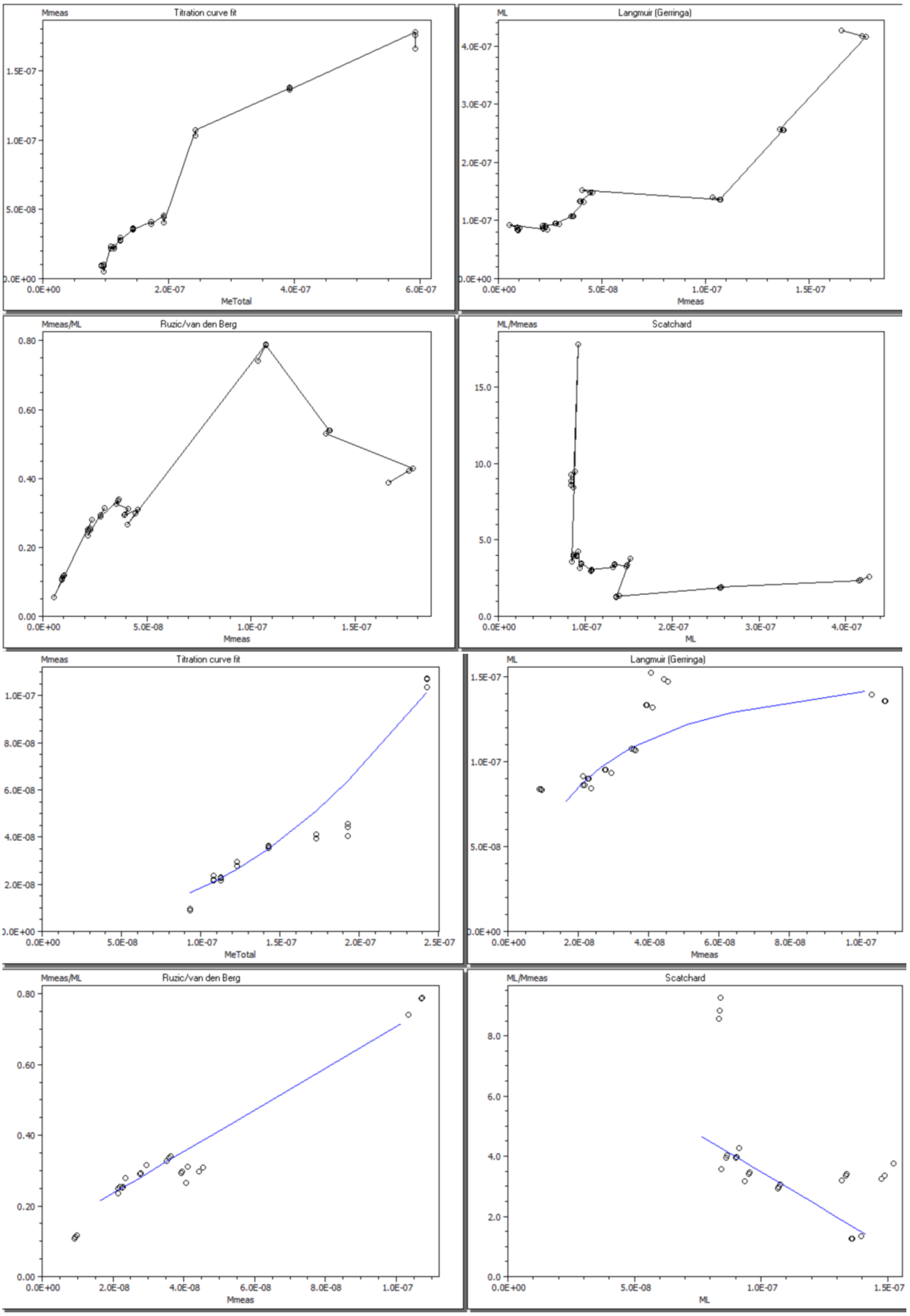

Good fit examples

Sample SO242_196ROVPC28_16-18cm

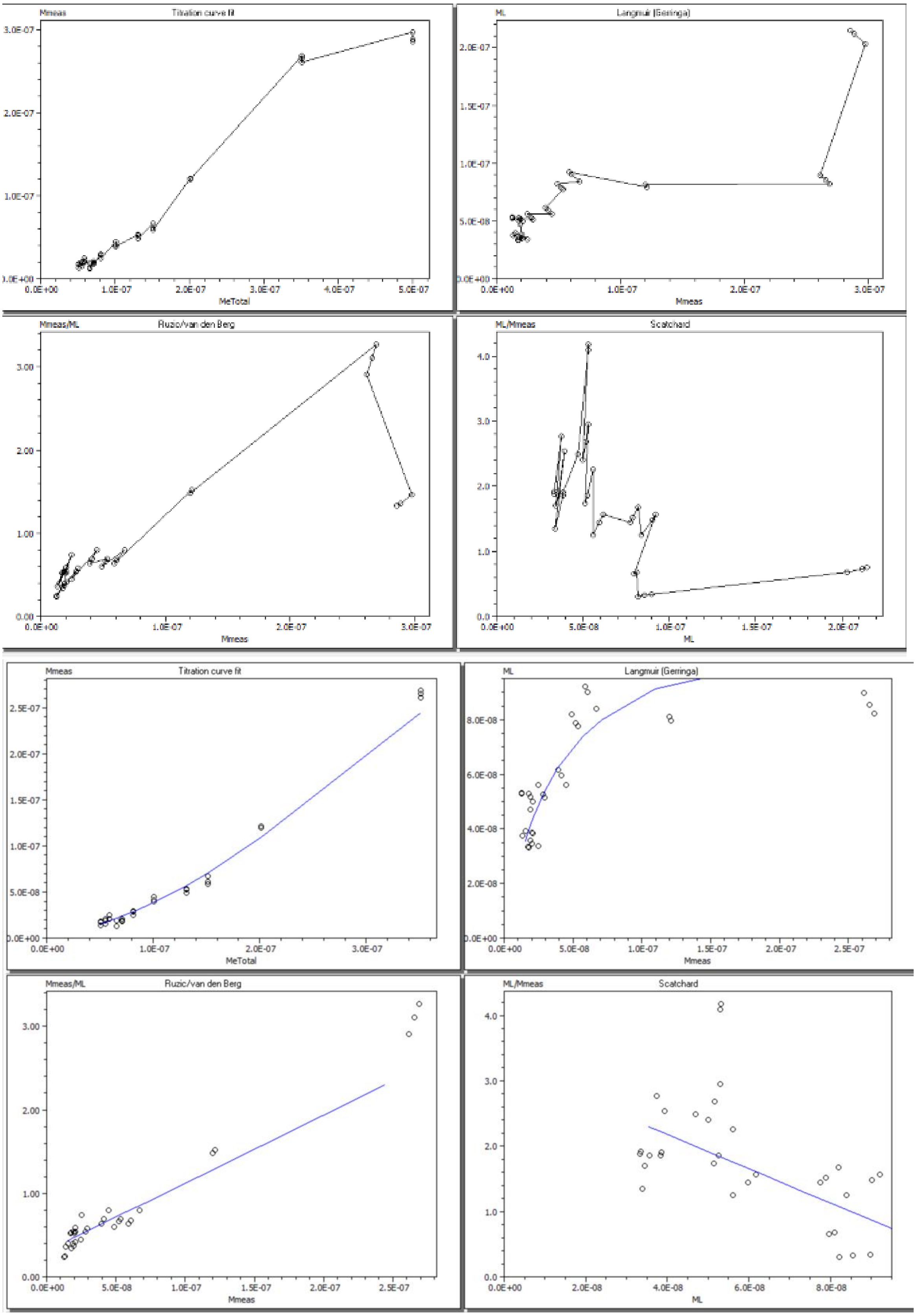

Sample SO242_146ROVPC28_BW

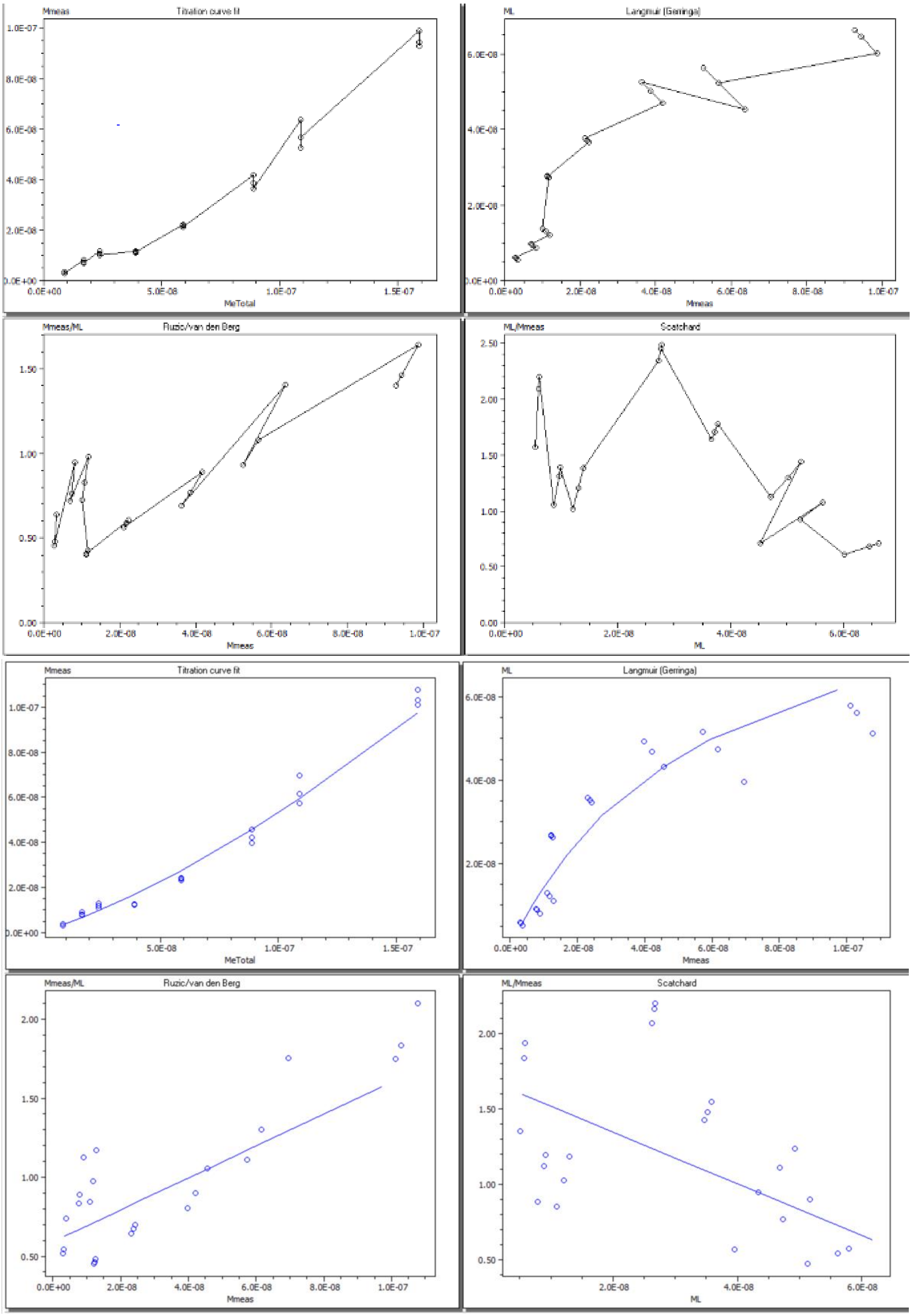

Supplement: Supplementary file 1 — Supplementary Information. [file 41598_2021_97813_MOESM1_ESM.pdf]
